# Engineering extracellular vesicles for ROS scavenging and tissue regeneration

**DOI:** 10.1186/s40580-024-00430-9

**Published:** 2024-06-26

**Authors:** Ahmed Abdal Dayem, Ellie Yan, Minjae Do, Yoojung Kim, Yeongseo Lee, Ssang-Goo Cho, Deok-Ho Kim

**Affiliations:** 1 Department of Stem Cell and Regenerative Biotechnology, Molecular & Cellular Reprogramming Center, Institute of Advanced Regenerative Science, Konkuk University, 120 Neungdong-ro, Gwangjin-gu, Seoul, 05029 Republic of Korea; 2 Department of Biomedical Engineering, Johns Hopkins University, Baltimore, MD 21205 USA; 3 R&D Team, StemExOne Co., Ltd., 307 KU Technology Innovation Bldg, 120, Neungdong-ro, Gwangjin- gu, Seoul, 05029 Republic of Korea; 4 Department of Mechanical Engineering, Johns Hopkins University, Baltimore, MD 21205 USA; 5 Department of Medicine, Johns Hopkins University School of Medicine, Baltimore, 21205 USA; 6 Center for Microphysiological Systems, Johns Hopkins University, Baltimore, MD 21205 USA; 7 Institute for NanoBiotechnology, Johns Hopkins University, Baltimore, MD 21218 USA; 8 Department of Neurology, Johns Hopkins University School of Medicine, Baltimore, MD 21205 USA

**Keywords:** Extracellular vesicles, Tissue regeneration, Reactive oxygen species scavenging, miRNA delivery, Biomaterial scaffold

## Abstract

Stem cell therapy holds promise for tissue regeneration, yet significant challenges persist. Emerging as a safer and potentially more effective alternative, extracellular vesicles (EVs) derived from stem cells exhibit remarkable abilities to activate critical signaling cascades, thereby facilitating tissue repair. EVs, nano-scale membrane vesicles, mediate intercellular communication by encapsulating a diverse cargo of proteins, lipids, and nucleic acids. Their therapeutic potential lies in delivering cargos, activating signaling pathways, and efficiently mitigating oxidative stress—an essential aspect of overcoming limitations in stem cell-based tissue repair. This review focuses on engineering and applying EVs in tissue regeneration, emphasizing their role in regulating reactive oxygen species (ROS) pathways. Additionally, we explore strategies to enhance EV therapeutic activity, including functionalization and incorporation of antioxidant defense proteins. Understanding these molecular mechanisms is crucial for optimizing EV-based regenerative therapies. Insights into EV and ROS signaling modulation pave the way for targeted and efficient regenerative therapies harnessing the potential of EVs.

## Introduction

Tissue regeneration is a multifaceted process compromising various phases, which are vital for cellular survival and functionality [1]. Every year, millions of people suffer from various kinds of injuries brought on by accidents, life-threatening illnesses, and other causes. Despite extensive investigations into the mechanisms of tissue regeneration at the cellular and molecular levels, inadequate recovery outcomes continue to affect a significant global population, posing a major obstacle for regenerative medicine [2]. Recent research has underscored the significant impact of the immune response on the quality of healing, including the restoration of organ structure and function [3, 4].

Extracellular vesicles (EVs), membrane-bound vesicles of varying sizes ranging from nanometer to micrometer, are secreted by diverse cell types [5]. The significant impact of EVs in cell-to-cell interaction has been evidenced by numerous studies [6–8]. EVs can be categorized into three main subtypes based on their biogenesis, source, and size: exosomes, apoptotic bodies, and microvesicles [9, 10]. EVs are found in various bodily fluids, such as blood, milk, urine, and saliva [11, 12].

Inconsistencies in terminology across research articles have led to ambiguity among scientists studying EVs [13]. To address this issue, the 2018 revision of the “minimal information for studies of extracellular vesicles 2018 (MISEV2018)” guidelines established “EVs” as the umbrella term for these particles [14]. These guidelines define EVs as naturally released cellular structures enclosed by a lipid bilayer and incapable of self-replication due to the lack of nuclear material.

EVs serve as biological carriers of various therapeutic molecules, including proteins, nucleic acids, lipids, microRNAs (miRNAs), and other RNAs. They engage with cellular receptors or target molecules, influencing cellular events and aiding in immune regulation to promote tissue regeneration [15]. EVs are therefore naturally occurring nanocarriers that contain functional cargo in phospholipid-bilayer nanovesicles for biomedical applications, offering advantages over traditional nanoparticle (NP)-based approaches [16]. EVs faster uptake into the cell, a longer half-life in circulation, and enhanced functionality with the targeted ligand in addition to their physical characteristics have been evidenced [17].

The quantity, composition, and functionality of EVs are influenced by factors such as their parent cell origin, the physiological or pathological state, and the microenvironment [18]. Stem cell-derived EVs, in particular, exhibit paracrine functions, influencing the adjacent or remote cells via the delivery of cell-modulating factors [19]. Their capacity to transport both hydrophobic and hydrophilic substances within the lumen or on their lipid bilayers, coupled with protection from enzymatic degradation, underscores their potential as therapeutic agents [20, 21]. Furthermore, EVs derived from stem cells exhibit targeted specific types of cells via their surface receptors [22–24]. Besides, EVs are adept at crossing biological barriers and accumulating in various organs [22, 25]. EVs have outstanding biocompatibility, stability, and biodegradability, along with low immunogenicity [26]. This low immunogenicity is attributed to the lower expression of surface transmembrane proteins, such as major histocompatibility complex (MHC) compared to that of the host cell-derived vesicles [27]. The expression of specific surface proteins in the recipient cells and proteins in EVs determines the appropriate mechanism for EV internalization [28]. EVs have unique in vivo capabilities such as enhanced permeability and retention, because of their small size and minor negative zeta potential [29]. In sum, EVs are promising candidates for promoting tissue regeneration and triggering specific immune responses.

EVs have emerged as promising drug delivery agents, however, challenges persist in effectively loading RNA and protein into EVs and delivering them to specific target sites within the body [30]. While EVs homing ability possesses a great deal of therapeutic potential, in vivo studies have revealed insufficiency of their activity [28].

The clearance of natural EVs by the mononuclear phagocyte system or entrapment within the reticuloendothelial system can hinder their accumulation in the injured regions, hampering their regenerative effects [31]. Additionally, the insufficient capacity of the natural EVs to deliver the intended cargo to the targeted cells or tissues, prompted the development of various EV engineering approaches to enhance their therapeutic capabilities [24, 32].

The field of EV engineering has garnered significant attention for its potential as a target delivery method [33, 34], a diagnostic tool [35], and a therapeutic agent for diseases [36]. Furthermore, the capacity of EVs to mitigate oxidative damage has recently garnered interest with studies demonstrating their antioxidant activity in treating various diseases, such as wound healing [37], bone loss [38], osteoarthritis (OA) [39], hepatic injury [40], Parkinson’s disease (PD) [41], hyperglycemia [42, 43], ischemia-associated damage [44], brain injury [45], intervertebral disc degeneration [46], and colitis. Reactive oxygen species (ROS) scavenging activity plays a key role in the healing of chronic wounds [47, 48]. For various applications in regenerative medicine, utilization of nanomaterials with ROS-scavenging activity, such as antioxidant-conjugated nanomaterials enzyme-mimicking nanomaterials, and polymeric NP has been evidenced [49–51]. Additionally, various reports have demonstrated the beneficial effects of ROS signaling pathways on the regeneration of several tissues, such as skin, bone cartilage, and the heart [50, 52–54].

EVs derived from mouse inner ear stem cells, mesenchymal stem cells (MSCs), and human hepatic progenitors have been shown to effectively mitigate oxidative damage both in vivo and in vitro [55–60]. These EVs are enriched in antioxidant enzymes such as glutathione peroxidase, superoxide dismutase 1 (SOD1), SOD2, catalase (CAT), and others further emphasizing their role in redox regulation and tissue repair [61].

In this perspective, we review recent advances in EV engineering strategies and their mechanisms that are involved in tissue regeneration. Additionally, we elucidate the interplay between EVs and ROS modulation, a critical aspect of EV-mediated therapeutic applications in tissue regeneration. We also outline the recent advancements in EV engineering methods and their application of engineered EV tissue regeneration, focusing on bone, muscles, and the peripheral nervous system (PNS). With further improvements in EV quality, quantity, and engineering techniques, the engineered EVs hold promise for robust therapeutic activities that may revolutionize their therapeutic applications in tissue regeneration.

## Approaches to enhance the potential of EV-mediated tissue regeneration

The application of natural EVs in research presents significant challenges owing to their diversity and heterogeneity, arising from the varying types and phenotypes of their source cells, thereby resulting in the secretion of several types of EVs [62]. Specifically, from the perspective of ROS scavenging for tissue regeneration, natural EVs demonstrate limited capacity for direct ROS scavenging and are often not efficiently delivered to specific tissues, being rapidly cleared from the body instead. To address these challenges, various strategies have been devised, including the loading of EVs with ROS-scavenging materials surface modifications to enhance tissue-specific delivery efficiency. Additionally, indirect engineering approaches involve subjecting the EV-secreting cells to physical or chemical stimuli or utilizing various scaffolds to maximize therapeutic effects.

The necessity for engineering EVs arises from two principal perspectives. Firstly, concerns revolve around the rapid clearance of EVs by the liver and kidneys, resulting in a circulating half-life of under one hour, posing significant hurdles for regenerative therapeutic applications [63]. The short circulation time hinders EVs from reaching target organs promptly, impeding their regenerative potential. To overcome this, various methods have been explored to ensure efficient delivery to target organs, including encapsulating EVs within a decellularized extracellular matrix (ECM) to restrict them to the target site [64, 65], or artificially modifying the surface of EVs for targeted tissue delivery, have been explored [66].

Secondly, there is a pressing need to load specific therapeutic agents into EVs for the treatment of various diseases, such as guide RNA (gRNA) and Cas9 protein for genome editing [67], ROS scavengers for combating oxidative stress [68], or other drugs. However, EVs are enveloped in a phospholipid bilayer, approximately 4–5 nm in size, which impedes the permeation of hydrophilic substances or those larger than 500 Da making the internalization of drugs a complex task. Despite the necessity, it is challenging to load drugs inside EVs, and a variety of loading methods have been investigated. There are diverse methods for modifying EVs including chemical, genetic, or physical approaches. Each of these methods has its advantages and disadvantages, and a prudent choice is required based on the target disease.

### Chemical modification

Chemical modification of the EV surface through induced chemical bonding stands as a widely employed strategy. This approach enables the introduction of various biomolecules, including peptides, nanobodies, and antibodies, which specifically target marker proteins. Such a strategy provides a reliable way to introduce desired substances onto the surface without causing physical denaturation of the EV membrane bilayer. It is a common technique due to its effectiveness and minimal impact on the structural integrity of EVs. However, there is a risk of altering the intrinsic properties of EV surface proteins through these chemical reactions, and prolonged reaction times at room temperature (RT) can lead to issues such as EV loss. Furthermore, since most chemical reactants cannot penetrate the EVs, there are limitations to loading therapeutic substances inside. One of the common bioconjugation methods is utilizing *N*-ethyl-*N*′-(3-(dimethylamino)propyl) [69].

Additionally, bio-orthogonal modification techniques involve engineering the cell membrane of EV-producing cells to introduce alkyne groups, which can then be targeted with azide-modified substances. This chemical conjugation method is suitable for introducing aptamers onto the surface of EVs. Aptamers are synthetic oligonucleotides that adopt specific structures and selectively bind to targets [69]. With their broad spectrum of targets and excellent binding affinity and specificity, they were extensively used for molecular probes [70, 71]. The integration of aptamers with exosomes represents a novel avenue in EV-related research [70, 72, 73]. Namely, the antifibrotic effects of EV-delivered aptamer S58 were evaluated in human fibroblast and a rat glaucoma filtration surgery model, demonstrating superior results with reduced fibrosis and improved filtering bleb retention [74]. Another study developed aptamer-functionalized EVs, utilizing the diacyllipid–aptamer conjugates as targeting ligands, for cell-type-specific molecular therapeutic delivery. These aptamer-modified EVs showed lower cytotoxicity and more efficient cellular uptake than their unmodified counterparts [75].

#### Bioconjugation chemistry (EDC/NHS and click chemistry)

Chemical reactions typical of bioconjugation, such as the 1-ethyl-3-(3-dimethylaminopropyl) carbodiimide (EDC) and N-hydroxysuccinimide (NHS), maleimide reactions, and Click chemistry are fast methods and proceed well at neutral pH. The methodological approach typically involves purifying EVs first and then chemically bonding targeting molecules to the amine groups, carboxyl groups (such as those from glutamic acid or aspartic acid), or thiol groups of exosome membrane proteins [76, 77]. To minimize the exposure of EVs to RT, most procedures involve pre-reacting the target molecule with EDC/NHS or maleimide before the secondary reaction with the EVs. However, the direct reaction between the targeting molecule and EV membrane proteins can be impeded due to steric hindrance, leading to the use of linkers such as polyethylene glycols (PEGs) larger than 2000 Da, and the PEG has a functional group for further cross-linking to target molecules [78]. Among these, the chemical bonding of streptavidin to the EV terminus and biotin to the targeting molecule is a widely chosen method due to the high-affinity binding of streptavidin and biotin [79]. However, this chemical bonding approach has the disadvantage that the surface-bound drugs on EVs may easily degrade in the acidic environment encountered after cellular uptake, posing a risk of degradation.

#### Bio-orthogonal modification

Bio-orthogonal modification is a technique inspired by cell membrane engineering that primarily involves supplying cells with lipids tagged with an externally clickable functional group (alkyne) [80]. These lipids naturally incorporate into the EVs during their biogenesis. Subsequently, EVs are purified and acquired, and then mixed with a targeting molecule that has been introduced with an azide group, resulting in surface modification. The advantage of this method is that once the alkyne group is introduced onto the EV surface, the click reaction occurs quite easily, minimizing the exposure time of EVs to RT and specifically allowing the modification of lipid components [81].

Since its introduction in 2015 [82], this approach has been utilized for various EV modifications [83]. On the other hand, there are cost-related drawbacks as the lipids with alkyne groups are expensive, and not all alkyne-functionalized lipids may be intercalated into the EVs, which can be a disadvantage compared to other chemical bonding methods [84]. Additionally, there is a need to optimize the timing for EV extraction since alkyne-introduced lipids naturally degrade over time, which necessitates identifying the optimal time for EV harvest.

### Genetic modification

The genetic modification method involves altering the genome of cell lines that produce EVs to equip them with desirable properties. This is achieved by introducing external genes into specific cells. The methods for introducing external genes include using viral and non-viral substances to create a gene-modified cell line either in the cell genome or episome [85]. This approach can produce engineered EVs naturally during the biogenesis process without additional steps. However, establishing such cell lines is costly and labor-intensive.

It is also challenging to control the degree of modification for each EV, and continuous genetic changes need to be monitored during long-term culture and production. The applications of this genetic modification method include overexpressing proteins within the cell to be naturally incorporated into the EVs [86], attaching desired proteins to the surface of genetically modified EVs [87], and engineering EV surface proteins to display peptides that interact with specific target tissues or cells, thereby enhancing delivery efficiency [88].

#### Protein engineering for EV-mediated tissue targeting

There is a method for overcoming the short clearance time of EVs in the body by genetically fusing specific peptides that can target specific tissues to the surface of EVs. These targeting peptides can vary depending on the target tissue. Commonly used fusion proteins include tetraspanin family proteins like CD9, CD63, and CD81, known as EV markers. However, these proteins are not suitable for tissue targeting as both their N- and C-termini face inside the EVs. Instead, most studies primarily use lysosome-associated membrane protein (LAMP)2B, which is an EV membrane protein. An interesting study has demonstrated the targeted delivery of *GAPDH* siRNA to the mouse brain using EVs purified from dendritic cells engineered with LAMP2B integrated with rabies viral glycoprotein (RVG) peptide that is associated with the central nervous system (CNS) [88]. The main issue with these surface-targeting peptides is degradation due to low pH inside the endosome during EV biogenesis. To address this, in 2019, Hung introduced a method that fuses a glycosylation motif at various sites to protect the targeting peptide from lysis and boost the expression level of LAMP2A in cells and EVs [66].

#### EVs engineering via loading of synthetic protein

EVs for protein loading through utilizing the optically reversible protein-protein interactions (EXPLORs), an optogenetic tool, which showed a successful application in loading target proteins inside EVs for the intracellular delivery of the cargo proteins [87]. In this study, they used a combination of synthetic proteins such as photoreceptor cryptochrome 2 (CRY2) and CRY-interacting basic-helix-loop-helix 1 (CIB1) proteins, which were fused with the tetraspanin protein CD9 (an EV marker protein) and a cargo protein, respectively and illumination of the blue light. After the cargo proteins have been packed into the EVs through endogenous biogenesis, they can be released into the intraluminal space of the EVs and effectively delivered to the cytosolic compartment of target cells by separating from CD9-conjugated CIBN (a truncated form of CIB1) by eliminating the illumination source. This innovative approach allowed for the packaging of proteins into the EVs by exposing them to light during EV biogenesis [87]. This method, which relies on blue light exposure, has the advantage of securely packaging target proteins inside EVs.

However, it has limitations in loading proteins larger than 40 kDa and in the types of proteins that can be loaded due to the weaker affinity of optogenetics structural changes and the free movement of proteins in the cytosol before loading.

#### Overexpression of target proteins

In this approach, the target proteins are overexpressed in the specific cells that are used for EV isolation and the purified EVs are loaded with the target proteins. Some methods demonstrated the overexpression of target proteins in specific cell lines leads to their stochastic incorporation into EVs. It has been reported that overexpression of stromal cell-derived factor 1 (SDF-1), a factor known for attracting stem cells, in MSCs, aids cardiac repair post-myocardial infarction [86]. The application of EVs from these SDF-1-overexpressing cells to a myocardial infarction model showed promising results in regenerating endothelial microvasculature [86]. Another study involved overexpressing the macrophage migration inhibitory factor (MIF) in MSCs and then observing myocardial repair. The study confirmed through animal experiments that EVs containing MIF helped in recovery by preventing early inflammation at the damaged site [89]. This method, while simple and not requiring complex systems, relies purely on a chance for protein loading into EVs, making efficient loading challenging. The balance between cell growth and programmed cell death in the cells that supply proteins might be disrupted by the overexpression of proteins. It also has the drawback of not being able to control the amount of protein loaded per EV. Below, Table 1 summarizes the methods for the engineering of the EVs, genetically or chemically.

**Table 1:** A Comprehensive Overview of EVs Engineering Techniques

Category	Purpose	Method	Material	Target	References
Bio-orthogonal modification	Drug loading	Metabolic glycoengineering for click chemistry	Tetraacetylated N-azidoacetyl-D-mannosamine, L-azidohomoalanine	B16F10 cell Horse radish peroxidase loading	[82]
Bio-orthogonal modification	Tissue targeting	Metabolic glycoengineering for click chemistry	N-azidoacetyl-d-mannosamine, dibenzocyclooctyne	Rheumatoid arthritis, tumor	[83]
Genetic modification	Tissue targeting	Plasmid transfection to EV-producing cell	LAMP2B, Rabies virus glyco peptide	Mouse brain	[90]
Genetic modification	Tissue targeting	Glycosylation of EV	LAMP2B	Enhancing EV stability	[66]
Genetic modification	Drug loading	Optogenetic for protein loading into EV	CD9, CRY2, CIBN	Cre delivery to Brain	[87]
Genetic modification	Drug loading	Overexpression of cargo protein	SDF1	Myocardial infarction	[86]
Bioconjugation chemistry	Imaging	EDC/NHS reaction for further click chemistry	EDC/NHS, 4-pentyonic acid	4T1 cell crosslinking Azide-Fluor 545	[76]
Bioconjugation chemistry	Tissue targeting	Streptavidin and biotin	DSPE-PEG-biotin	Periodontal defect	[79]

*Abbreviations* ADMSC, adipose-derived mesenchymal stem cell; MSC, mesenchymal stem cell; BMSC, bone marrow-derived stem cell; GMSC, gastric stem cell; UCMSC, umbilical cord mesenchymal stem cell; EDC/NHS, 1-ethyl-3-(3-dimethylamino) propyl carbodiimide, hydrochloride and N-hydroxysuccinimide; LAMP2B, lysosome-associated membrane protein 2B; CRY2, cryptochrome 2; DSPE-PEG-biotin, 1,2-distearoyl-sn-glycero-3-phosphoethanolamine-N-[biotinyl(polyethylene glycol)]

### EV cell source modification

While MSC-EVs have undergone thorough investigation as a potential cell-free therapy for various diseases, hurdles including low EV yield, heterogeneity, and limited targeting capacity hamper their clinical translation [91]. Therefore, pre-conditioning or pre-stimulation of the parental cells could represent a promising strategy to enhance paracrine activity and augment the quantity and quality of EV production. In this section, we provide a brief overview of potential strategies to optimize cell conditions for obtaining high-quality EVs.

#### Three-dimensional (3D) cell culture

MSC culture is carried out using a 2D culture platform, or utilizing static adherent cultures, in the presence of human-originated supplements or fetal bovine serum (FBS). However, notable distinctions exist between 2D and 3D culture platforms, including nutrient release, medium composition, and biological activity. In 2D cultures, MSCs gradually lose their native cytoskeleton organization, potentially compromising their ability to proliferate and differentiate, thereby impacting the therapeutic qualities of their EVs [92, 93]. Consequently, conventional 2D culture methods often yield suboptimal EV production efficiency [93, 94]. Hence, there is an imminent need to obtain stem cells with enhanced stability and superior quality for future cell-free therapies, potentially surpassing the limitations of 2D cultivation.

Currently, two main types of 3D culture platforms are utilized: material-dependent and material-free cultures. Material-free cultures involve the formation of spheroids through the cell aggregation process. The most common techniques include hydrogel-assisted 3D culture [95, 96], agitated culture [97], scaffold-free suspension cultures [98], and 3D spherical spatial boundary culture [99].

Various models represent the material-assisted 3D culture platform, such as fibrous scaffolds [100], native ECM scaffolds [101], hollow fiber bioreactors [102], quantum cell expansion systems [103], and computer-controlled bioreactors [104]. This platform facilitates close, dynamic interaction with the culture medium, fostering the formation of intricate 3D structures. Notably, employing a 3D culture system yields a considerable number of MSCs [93, 97, 99] and a high yield of MSC-EVs [96, 102, 105], attributed to favorable void structure, surface activities, mechanical strengths, and biocompatibility. For example, utilizing a hollow fiber bioreactor-based 3D culture system significantly increased the total amount of MSC-EVs by approximately 19.4 times compared to 2D culture [102, 106].

The application of 3D-derived EVs (3D-EVs) offers distinct benefits in various injury repair processes, notably in promoting angiogenesis, migration, and proliferation of endothelial cells [107]. Previous reports have demonstrated the robust therapeutic potential of 3D-EVs in the recovery of cisplatin-induced acute renal injury [105] and the disorders affecting the central nervous system [104, 108, 109]. For instance, following severe brain damage in rats, 3D-EVs localized within collagen scaffolds could enhance neurovascular remodeling and functional recovery. Utilizing a 3D dynamic culture platform supplemented with exogenous TGF-β3 for Wharton’s Jelly-derived MSCs ( WJ-MSCs), our research group has obtained protein cargo-enriched Ta3D-WJ-MSC-EVs with high therapeutic efficacy that have shown enhanced in vitro migration and marked healing in the in vivo excisional wound model [110]. Moreover, we demonstrated the in vivo therapeutic potential of Ta3D-WJ-MSC-EVs against the inflammatory reaction and bladder dysfunction in an experimental interstitial cystitis/bladder pain syndrome (IC/BPS) [111].

#### Physicochemical stimuli

Environmental parameters play a pivotal role in modulating physiological conditions that not only facilitate the production of EVs but can also enhance their therapeutic properties [106, 112, 113]. The major category is physical cues, which can be further delineated into mechanical, acoustic, and electrical stimuli. Cellular response to mechanical stimuli plays a fundamental role in cellular communication and the microenvironment [114], with altered mechanical stimuli known to trigger increased EV secretion [113]. For instance, a study on skeletal muscle cells revealed that mechanical strain augments total EV production and that EVs demonstrate the ability to enhance the proliferation and myogenic differentiation of naïve C2C12 cells [115].

Moreover, several investigations have also demonstrated improved drug loading into EVs following sonication [116–118]. Most of the loaded cargo consists of anti-tumor drugs, and the utilization of EVs significantly enhances targeting specificity. For instance, a smart nanosonosensitizer was engineered by conjugating sinoporphyrin sodium (DVDMS) onto tumor cell-derived EVs, exhibiting high stability, homotypic tumor targeting, and ultrasound-responsive drug release for enhanced sonodynamic therapy (SDT) [116]. Furthermore, evidence suggests that electrical stimulation under certain conditions can have effects such as cardioprotection [119], improving peripheral neuropathy [120], and spinal cord repair [121].

Besides physical stimulation, biochemical methods offer avenues for preconditioning the EV cell sources. Key parameters requiring modulation include oxygen level [122–124], glucose concentration [125–127], and acidity [128, 129]. Numerous research studies have demonstrated that conditions such as hypoxia, glucose starvation, and low pH, traditionally thought to be detrimental to cell growth, can paradoxically stimulate EVs release while activating pathways associated with stress and survival. Furthermore, other chemical agents, such as melatonin pre-treated MSC- EVs, have shown promise in enhancing angiogenesis for the treatment of chronic kidney disease (CDK) [130] and mitigating acute liver ischemia-reperfusion injury [131].

Furthermore, cytokines, as inflammatory-related molecules, have been identified as key regulators of EV production [132–136]. The introduction of specific cytokines can initiate various downstream mechanisms in target cells, as evidenced by a study demonstrating a significant increase in the anti-inflammatory efficacy of IL-1β-primed MSC-EVs in osteoarthritic cells. This impact is facilitated through miR-147b, leading to the suppression of the NF-κB pathway [137]. Various miRNAs, such as mi146a, are upregulated in the murine sepsis models and concentrated into EVs upon IL-1β treatment [138]. Moreover, evidence suggests, in the presence of IL-1β, EV miR-21 can efficiently stimulate macrophages towards M2 polarization, both in vitro and in vivo, resulting in therapeutic effects against sepsis [139]. Similarly, TNF-α pretreatment has been shown to enhance therapeutic efficacy of T-hUCMSC-EVs for acute liver failure by suppressing the breakdown of the Golgi structure, thereby preventing the recruitment and activation of NLRP3 in macrophages [140]. Notably, the authors detected the high expression of miRNA-299-3p in T-hUCMSC-EVs, a key mediator of their anti-inflammatory activity in acute liver failure [140]. Similarly, pretreatment of gingival tissue-derived MSCs (GMSCs) with TNF-α leads to the production of EVs with potent capacity for treating periodontitis by modulating inflammation and osteoclastogenesis [141]. This activity is attributed to miR-1260b, which targets the Wnt5a-associated RANKL pathway. It overall increases the EVs secretion and CD73 expression level, promoting anti-inflammatory M2 macrophage polarization [141]. TNF-α pre-treated MSCs also exhibit increased antifibrotic effects on fibroblasts to treat urethral stricture, with miR-146a absorbed by the EVs [142]. These findings collectively showcased the versatility of cytokine-induced modifications in tailoring EV capacity for diverse therapeutic applications.

For obtaining high-purity EVs, a novel approach demonstrated the production of WJ-MSC-EVs on a large scale in just 15 min utilizing Noxa-derived peptide, eMTDΔ4 [143]. In this study, cells were dissociated and then treated with eMTDΔ4 in a chemically defined sucrose buffer and incubated on the orbital shaker. WJ-MSCs exhibited a significant (approximately 30-fold) increase in the number of EVs with superior purity (about 45-fold). After 48 h, these EVs surpassed natural EVs extracted from the culture media in terms of their capacity for immunomodulation and regeneration [143].

In summary, the 3D culture platform, in addition to the pre-stimulation of cells with cytokines, peptides, physical stimuli, and growth factors, holds promise in significantly enhancing the yield, purity, and therapeutic capacity of EVs.

### EV conjugation with biomaterial scaffolds

Besides engineering the EVs, the integration of EVs with biomaterial scaffolds marks a compelling convergence of two powerful regenerative strategies [144, 145]. EV-conjugated scaffolds leverage the advantages of both components: the structural and delivery capabilities of the scaffold and the regenerative potential of EVs. It could potentially overcome the disadvantages of the short retention of EVs after being introduced to human bodies [145]. For instance, hydrogel, as a 3D network of hydrophilic polymers, is a model candidate for various needs of tissue repair as it affords a nurturing microenvironment and therapeutic agents [146–149]. One study focused on the development of antibacterial polypeptide-based hydrogel loaded with adipose-derived mesenchymal stem cell exosomes (AMSCs-exo) that help in the responsive sustained release of AMSCs-exo [150]. AMSCs-exo demonstrated a robust capacity for the healing of chronic wound healing and skin regeneration in diabetic patients. Compared to using AMSCs-exo or the hydrogel alone, the combined hydrogel demonstrated superior diabetic wound healing outcomes, even leading to skin appendage regeneration and reduced scarring [150].

**Fig. 1:**
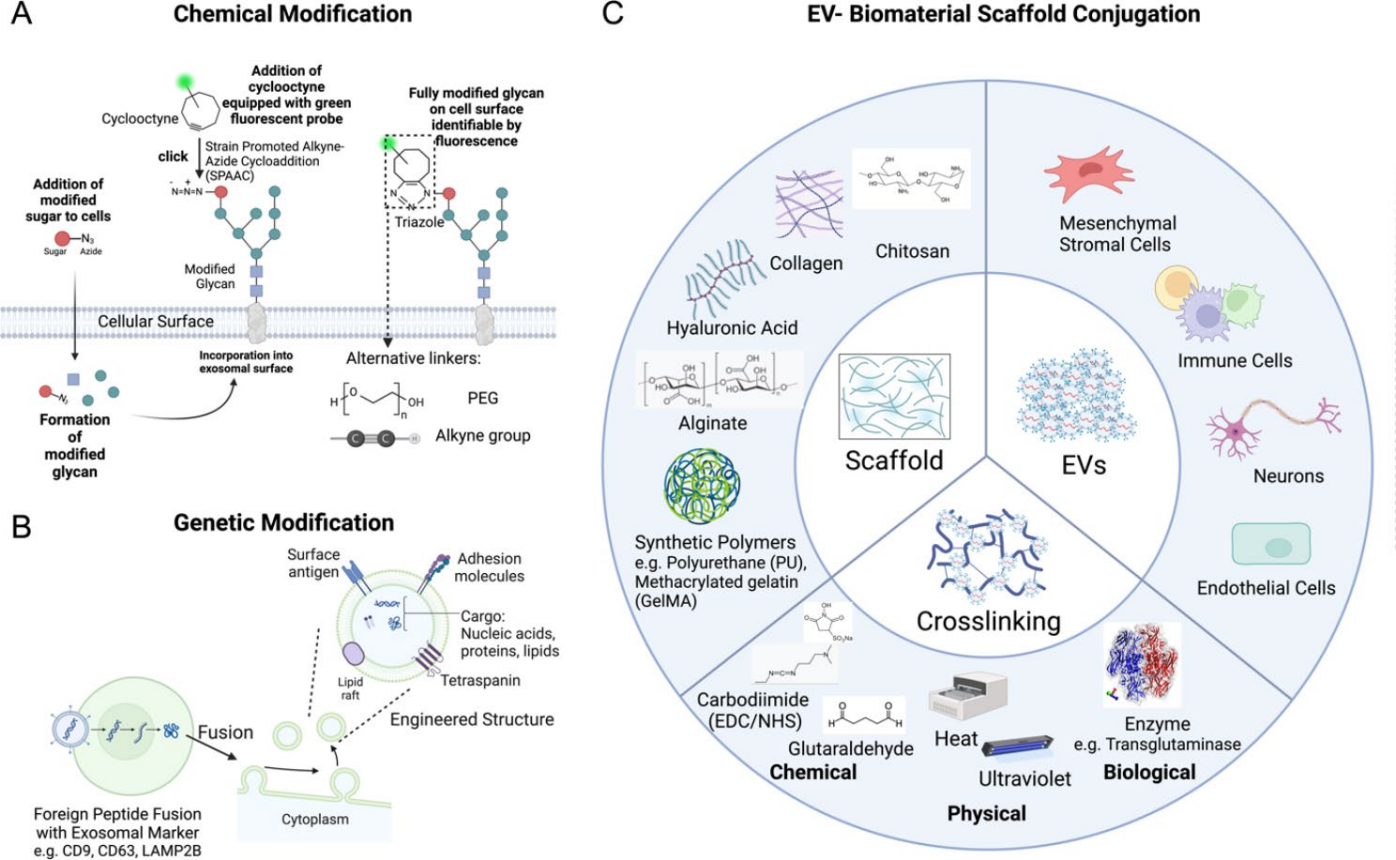
Schematics illustrating the different EV engineering methods. (**A**) EV engineering via chemical modification, with an emphasis on click chemistry. Synthetic linkers such as polyethylene glycol (PEG) and alkyne groups are incorporated. (**B**) EV engineering via genetic modification. In this case, the genetic materials encoding foreign peptides are introduced and expressed with EV markers. It results in modified surface antigens, tetraspanin, cargos, etc. (**C**) EV engineering via conjugating with biomaterial scaffolds. Examples of scaffold types, EV cell sources, and crosslinking methods are enumerated. This figure was created with BioRender.com, accessed on March 22nd, 2024

**Fig. 2:**
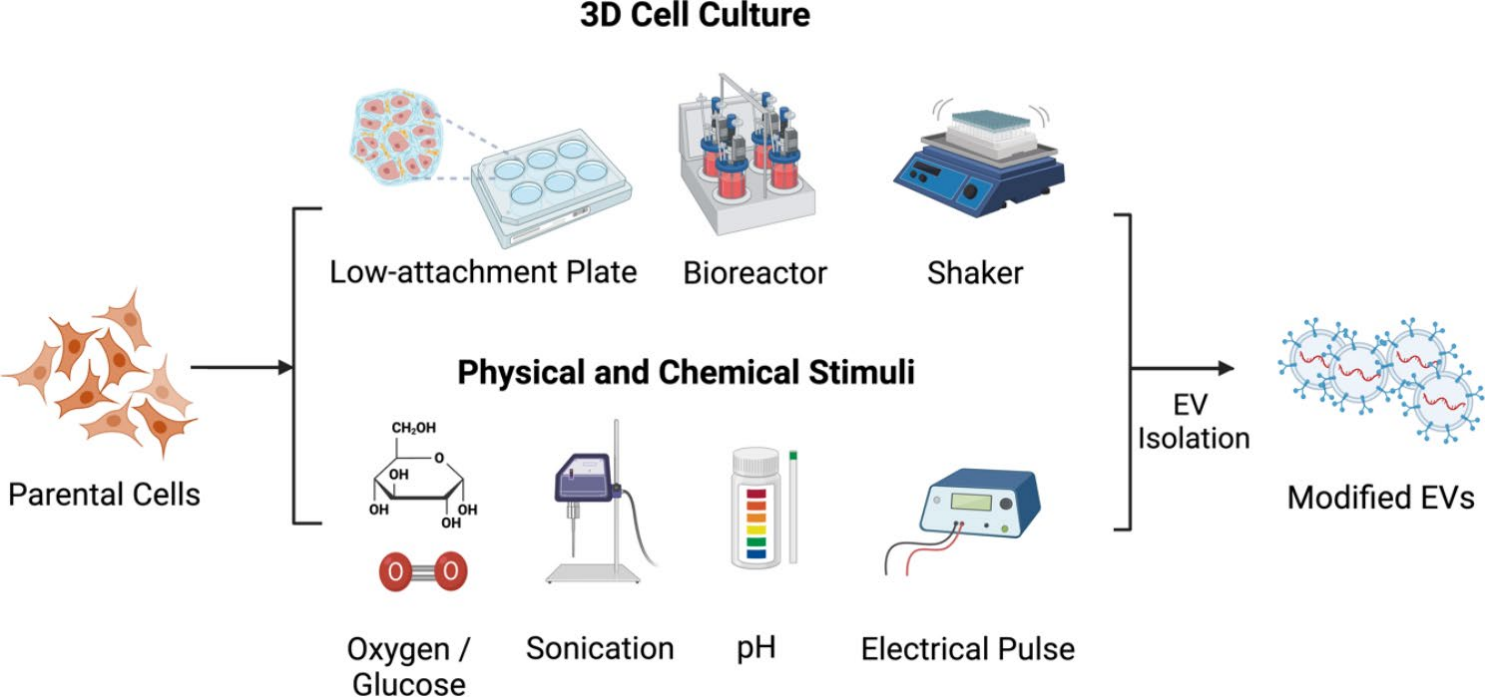
Schematics depicting the procedure for modification of EV cell source to enhance the quality and yield of purified EVs. The two approaches here are either changing the culture condition or introducing foreign stimuli. This figure was created with BioRender.com, accessed on March 29th, 2024

Another research on the 3D MSC-Exo demonstrated that, when conjugated with the GelMA (GelMA) microneedle patch, the results seem promising as the combination reduces inflammation and scarring for severe spinal cord injuries [151]. Another study purified the hucMSC-Exos and conjugated it with a PEG-based hydrogel composed of silk fibroin (SF), coralline hydroxyapatite (CHA), and glycol chitosan (GCS). In the rat femoral condyle defect model, bone repair was promoted [152]. Despite the diverse elements and structural variations in the hydrogel-exosome system, these investigations consistently reveal promise for the potential of this integrated therapeutic approach. A summary of the related studies of these EV-scaffold constructs is shown in Table 2 below. Collectively, this chapter explored the four major types of EV engineering methods for tissue regeneration. Figures 1 and 2 are schematic diagrams that illustrate the main EV engineering approaches in detail.

**Table 2:** Summary of Studies on Biomaterial Conjugated EVs

Disease Model	EV sources	Scaffold	Therapeutic effects	References
Myocardial infarction (rat)	ADMSC	Polyurethane-modified gallic acid (PUGA)- decellularized ECM	Reducing fibrosis and oxidative stress	[153]
Myocardial infarction (mouse)	MSCs	Fibrin	Smaller infarct size, more viable cardiac tissue	[154]
Myocardial infarction (rat)	ADMSC	Polyurethane (PU)-calcium peroxide (CPO)-Collagen	Decreasing scar and oxidative stress, improving cardiac function	[155]
Diabetic wound (rat)	BMSC	Cryogenic decellularized small intestinal submucosa (SIS)- mesoporous bioactive glass (MBG)	Improving vessel growth, collagen deposition, accelerated healing	[156]
Diabetic wound (rat)	GMSC	Chitosan/silk hydrogel	Promote healing of skin defects, higher collagen and microvessel composition	[157]
Bone defects (rabbit)	Serum	3D printed Strontium-Titanium	Accelerated bone repair and vascularization	[158]
Bone defects (rat)	ADMSC	Silk Fibroin	Integration of scaffolds improved osteogenic differentiation	[159]
Bone defects (rat)	Endometrial MSC	Hydroxyapatite (HA)	Enhancing osteogenesis and angiogenesis	[160]
Nerve injury-induced pain (rat)	UCMSC	Alginate	Antinociceptive, anti-inflammatory and pro-neurotrophic	[161]
Osteochondral defects (rat)	ADMSC	Methacrylated gelatin (GelMA)-ECM	Cartilage and bone regeneration	[162]

*Abbreviations* ADMSC, adipose-derived mesenchymal stem cell; MSC, mesenchymal stem cell; BMSC, bone marrow-derived stem cell; GMSC, gastric stem cell; UCMSC, umbilical cord mesenchymal stem cell; ECM, extracellular matrix

## EV as ROS-scavenging agent: therapeutic applications and modes of actions

### ROS and EV biogenesis

Oxidative stress is a pathophysiological process that is characterized by an imbalance between the antioxidants and pro-oxidants, or “a disruption of redox signaling and control” as described by Jones [163]. Oxidative stress impacts numerous biological functions such as cellular proliferation, immunological responses, steroidogenesis, aging, and cognition. However, an excessive amount of oxidant challenge leads to oxidative damage, triggering DNA damage, insulin resistance, and cardiovascular diseases [163–167]. Cellular organelles, including mitochondria, the endoplasmic reticulum (ER), microsomes, and peroxisomes are the major sources of intracellular ROS. Additionally, NADPH oxidase (NOX) complexes in cell membranes are implicated in ROS production [168, 169].

ROS play a crucial role in modulating the biogenesis and secretion of EVs. For instance, platinum NP-mediated high intracellular ROS levels boost EV biogenesis and secretion in human lung epithelial adenocarcinoma cancer cells [169]. Oxidative stress-mediated inhibition of lysosomal activity increases the cellular EV yield [171, 172], while mechanical injury-associated oxidative stress enhances EV secretion in lens epithelial cells [173]. Moreover, ROS generated due to the activation of the Ca^2+^-NOX5 (NAD(P)H oxidase 5) (NOX5) signaling axis, enhances EV release and vascular calcification [174]. In human retinal astrocytes (hRACs), tert-butyl hydroperoxide (tBHP) treatment increases both oxidative stress and autophagy, increasing the size of EVs without altering their composition [175].

EVs exhibit robust antioxidant activity by activating key antioxidant enzymes including glutathione peroxidase (GPX) [176]. Additionally, EVs play a role in suppressing ferroptosis and reducing ROS-associated neuronal cell injury [177]. MSC-EVs alleviate cognitive impairment by suppressing hippocampal ferroptosis in dNCR-aged mice, mediated by stimulating heme oxygenase-1 (HO-1), silent information regulator 1 (SIRT1), and factor nuclear factor-erythroid 2-related factor 2 (Nrf2) signaling pathways [178].

Human retinal pigment epithelium cells release more EVs containing greater amounts of VEGFR protein and mRNA when exposed to oxidative stress induced by treatment of 80 mM ethanol (EtOH) [179]. Notably, the increasing EtOH concentration over 200 mM impacts EV yield. Moreover, the abundance of EVs carrying Bax, Bcl2, and Atg12 is differentially influenced by varying EtOH concentrations [180].

Endogenous ROS induced by homocysteine (Hcy) drives the release of EVs encapsulating inflammatory cytokines [171]. In podocytes, the endogenous overproduction of ROS following Hcy activation reduces the release of lysosomal Ca^2+^ through the transient receptor potential mucolipin 1 (TRPML1) channel [171]. Moreover, TRPML1 inhibition enhances EV secretion by impeding Ca^2+^-dependent lysosome trafficking and subsequent lysosome-multivesicular bodies (MVB) interactions [171, 181]. In esophageal squamous cell carcinoma cells, it has been demonstrated that ROS-related mammalian target of rapamycin (mTOR) inhibition is augmented by treatment with the isothiocyanate sulforaphane (produced from cruciferous vegetables), leading to disturbance in the glutathione (GSH)/ oxidized GSH (GSSG) balance. Consequently, cytosolic TFE3 undergoes dephosphorylation, resulting in the emergence of aberrant lysosomes associated with TFE3, inhibiting MVB degradation and promoting increased EV release [172]. Under normal conditions, TFE3 is sequestered in the cytosol through association with the cytoplasmic chaperone 14-3-3 and phosphorylation by active mTOR [182]. TFE3, a key leucine zipper helix-loop transcription factor, translocates into the nucleus upon dephosphorylation, where it interacts with CLEAR elements in the promoters of multiple lysosomal genes, stimulating lysosome biogenesis [183].

ROS modulates the balance between the synthesis and degradation of autophagosomes, thereby regulating the degradation of MVBs [172, 175, 180, 182]. ROS signaling enhances autophagosome formation and suppresses autophagic reflux, leading to MVB degradation by autophagosomes and subsequent inhibition of EV release [175]. Moreover, ROS suppresses the fusion of autophagosomes and autolysosomes, further promoting EV secretion [175].

In airway epithelial cells, cigarette smoke extract (CSE), induces oxidative stress, thereby augmenting EV secretion [184]. In this study, oxidative stress-inducing compounds such as hydrogen peroxide (H_2_O_2_)and acrolein significantly impact cell viability. However, acrolein significantly enhances EV release, a phenomenon mitigated by thiol-reactive scavengers like GSH or N-acetylcysteine (NAC). Acrolein, but not H_2_O_2,_ modulates cellular GSH levels while concurrently reducing exofacial GSH levels, which rapidly revert after acrolein removal. In addition, a reduction in the exofacial GSH level is essential for the observed increase in EV release [184].

### Application of EV ROS-scavenging activity in tissue regeneration

EVs serve as inducers of various cellular signal transduction pathways because they encapsulate a variety of lipids, proteins, mRNA, miRNA, and other molecules [185]. Previous studies have demonstrated the potent capacity of MSCs, astrocytes, and neuronal progenitor cells to suppress oxidative stress-induced neuronal diseases [186, 187]. However, concerns regarding safety, ethical considerations, or national regulations restrict their clinical use [188]. Furthermore, the utilization of MSC-EVs, rather than MSCs themselves, may mitigate risks such as pulmonary embolism and chronic malignant transformation [189]. Notably, stem cell-derived EVs exhibit similar efficacy to their parent cells. The umbrella term " ROS” refers to a group of molecules or byproducts generated during the partial reduction of oxygen [190]. These highly reactive chemicals play a significant role in mediating inflammation, as reported in various studies [4]. Hydrogen peroxide (H_2_O_2_), superoxide anions (O_2-_), singlet oxygen (¹O-), and hydroxyl radicals (OH·) are key ROS elements that play critical roles in the regulation of various biological functions [191]. ROS concentration is maintained at a low physiological level, which is attributed to the dynamic balance via various signaling molecules [192]. The physiological ROS level is essential for activation of cellular signaling-mediated biological function. On the other hand, the disturbance of ROS generation or elimination leads to oxidative damage to nucleic acids, proteins, and lipids and pathological conditions such as inflammation [193, 194]. The ROS scavenging capacity of EVs plays a crucial role in tissue regeneration, either through direct modulation of ROS signaling pathways or delivery of antioxidant proteins [17, 55–61]. In this section, we will explore how the ROS scavenging function serves as a vital mechanism mediating EV-induced repair of various tissue injuries in vitro and in vivo.

#### Brain and neuronal disorders

In the brain tissues of PD patients, lower levels of SOD, CAT, oxidoreductase, and other antioxidants have been detected [195, 196]. However, the blood-brain barrier (BBB) hinders the delivery of the widely used antioxidants such as CAT [197]. The nanomaterials toxicity and the rapid drug clearance by the phagocyte system are the main issues of using the newly developed nano-delivery methods [198]. EVs, with their membrane layer, are hypothesized to overcome these challenges by penetrating the BBB and evading immune system clearance [134, 199]. Delivery of CAT mRNA via designer exosomes alleviated neurotoxicity in vitro and in vivo PD models [41]. In addition, the subcutaneous transplantation of exosomes has demonstrated robust in vivo ROS scavenging capacity against neurotoxic reagents such as 6-hydroxydopamine (6-OHDA) in mice.

For PD therapy, a research group has developed an ex vivo strategy for loading the potent antioxidant protein, CAT into EV using various methods, including permeabilization with saponins, incubation at RT, freezing/thawing cycles, extrusion, and sonication. This approach, efficiently enhances CAT loading capacity, prolongs the release, and protects against protease breakdown [199]. Using this approach, the authors verified the competent delivery of CAT-loaded EV into the neuronal cells and the brain of a 6-OHDA-induced mouse model, demonstrating marked anti-inflammatory and neuroprotective activity against oxidative stress-associated inflammation.

An interesting finding highlighted the antioxidant activity of umbilical cord MSC derived EVs (UC-MSC-EVs) and their therapeutic potential in vitro using H_2_O_2_-exposed hippocampal neurons and pilocarpine-induced seizures in an in vivo mouse model [188]. In both in vitro and in vivo models, MSC-EVs exhibited significant antioxidant activity, evidenced by enhanced ferric ion-reducing antioxidant ability, SOD, CAT, and GPX activities, reduced stress-associated molecular patterns, and DNA/lipid/protein oxidation, as well as diminished ROS generation. Additionally, MSC-EVs were found to be enriched in antioxidant miRNAs, namely miR-215-5p, miR-424-5p, miR-31-3p, miR-193b-3p, and miR-200b-3p. Mechanistically, the authors observed that the injection of AAV-Nrf2 attenuated the antioxidant capacity of MSC-EVs antioxidant capacity in seizure-mediated hippocampus injury, suggesting the involvement of the Nrf2 signaling pathway in the therapeutic potential of EVs against oxidative neuronal injury [188].

Another interesting study investigated whether ASC-Exo could protect motoneuron-like NSC-34 cells from oxidative damage, as well as an in vitro model of familial amyotrophic lateral sclerosis (fALS) (NSC-34 SOD1(G93A); NSC-34 SOD1(G37R); NSC-34 SOD1(A4V)) [200]. The authors added ASC-Exo to the culture medium along with H_2_O_2,_ effectively preventing apoptosis in NSC-34 and NSC-34 transfected cells and thereby enhancing cell survival.

EV-mediated miRNA delivery offers a promising approach to reducing oxidative stress via the PI3K/Akt signaling pathway. In hypoxia/reoxygenation (H/R)-injured endothelial cells (ECs), miR-132-3p-enriched BM-MSC-expos effectively decreased the level of ROS production, apoptosis, and tight junction breakage via directly inhibiting the expression of RASA1 and activating the phosphoinositide 3-kinase/AKT/endothelial nitric oxide synthesis (PI3K/AKT/eNOS) signaling pathway [201]. Following middle cerebral artery occlusion surgery, mice developed a focal ischemic stroke characterized by elevated ROS levels in cerebral microvascular endothelial cells. However, intravenous (i.v.) injection of BM-MSC-exos via the tail vein led to a reduction in ROS level, with effectiveness correlated with the amount of miR-132-3p delivered to cerebral microvessel cells. Interestingly, in vitro, blocking of the PI3K/AKT/eNOS signaling pathway partially reversed the impact of miR-132-3p-enriched BM-MSC-exos on reducing ROS generation in H/R-injured ECs [201].

Astrocyte-derived EVs that are enriched in miR-29a have been shown to effectively alleviate pyroptosis and mitigate apoptosis and oxidative stress. This effect was observed following oxygen and glucose deprivation in mouse microglia N9 models, as well as in rat models of brain ischemia-reperfusion injury (BIRI) [202]. The mechanism underlying these benefits is attributed to the restoration of miR-29a levels in the affected cells upon receipt of the EVs. miR-29a inhibits the expression of tumor protein 53-induced nuclear protein 1 (TP53INP1) by targeting its 3′ UTR, resulting in reduced apoptosis, malondialdehyde (MDA), inflammatory cytokines, and pyroptosis levels, as well as increased levels of SOD, GPX, and CAT and inactivation of a nuclear factor-kappa B/ nitrogen lipid regulator protein 3 (NF-κB/NLRP3) pathway.

A study revealed the potent activity of the amniotic fluid stem cells-derived exosomes (AFSC-Exos) in alleviating the Alzheimer’s disease (AD) phenotype in AD neuron primary culture by promoting cell viability and slowing down the progression of Aβ-induced neuronal death [203]. This activity attributed to the antioxidant activity of AFSC-Exos, as evidenced by the high expression levels of the antioxidant enzyme selenoprotein thioredoxin reductase 1 (TrxR1), TrxR2, SOD1, glutathione peroxidase, and GSH, which reduce ROS level in the neurons. Activation of the PI3K/Akt signaling pathway and concurrent suppression of NOX4 activity partially contribute to the antioxidant efficacy of AFSC-Exos [203]. In addition, a research report demonstrated the capacity of BM-MSC-Exos to transfer CAT and effectively restore basal neuronal ROS level elevated by amyloid-β peptide (AβOs) production [186]. Consequently, BM-MSC-Exos exhibited protective action on hippocampal neurons against AβOs-mediated synapse damage and oxidative stress. Pretreatment with a membrane-permeant-specific CAT inhibitor, 3-amino-1,2,4-triazole, abrogated the antioxidant activity of BM-MSC-Exos and the subsequent neuroprotective function.

The potent antioxidant capacity of the AD-MSC-Exo derived from TNF-α- and IFN-α-activated MSCs, has been demonstrated in a study where it suppressed chronic alcohol intake by 84% and attenuated ‘binge’ drinking following alcohol withdrawal when administered intranasally in rats [45]. This activity is attributed to the capacity of the exosomes to counteract the oxidative stress induced by alcohol in the hippocampus, as evidenced by a lower ratio of oxidized to reduced glutathione, along with their role in enhancing the expression of nuclear glutamate transporter 1 (GLT1), which reduces the inflammatory microglial population.

Moreover, a study revealed the protective effects of AD-MSC-Exos against radiation-induced brain injury by reducing oxidative stress, inflammation, and microglial infiltration, which is mediated by SIRT1 pathway activation [204]. This effect was abolished following treatment with the SIRT-1 inhibitor EX527.

#### Liver diseases

Chronic liver diseases, encompassing conditions like fatty liver disease, liver fibrosis, alcoholic liver disease, and viral hepatitis, are significantly influenced by increased oxidant stress [205]. Dysregulated intracellular oxidative stress is often associated with the progression of hepatocarcinogenesis [206], prompting extensive research into the therapeutic potential of antioxidants for liver diseases [207–210].

The in vitro and in vivo capacity of GPX1, a specific antioxidant enzyme found in hUC-MSC-Exo, has been demonstrated to ameliorate hepatic oxidant damage [40]. Administration of hUC-MSC-Exo, even at a low dose of 16 mg/kg body weight, via oral gavage or i.v. injection, exerted anti-apoptotic and antioxidant effects, preventing liver failure in mice exposed to the hepatotoxic compound carbon tetrachloride (CCl_4_). This protective effect is attributed to the exosome-mediated delivery of GPX1, which attenuates hepatic ROS levels and inhibits oxidative stress-induced apoptosis by suppressing the IKKB/NFkB/casp-9/-3 and upregulating B-cell lymphoma-2 (Bcl-2) and extracellular-regulated kinase 1/2 (ERK1/2) signaling pathways. Notably, the antioxidant and anti-apoptotic properties of hUC-MSC-Exo were abolished when GPX1 was knocked out in hUC-MSC, reaffirming the crucial role of GPX1 in their hepatoprotective activity. Furthermore, hUC-MSC-Exo exhibited superior antioxidant and hepatoprotective effects compared to the commonly used hepatoprotective drug bifendate (DDB) [211].

Similarly, hUC-MSC-EVs demonstrated potent in vitro and in vivo antioxidant and anti-apoptotic effects against hepatic ischemia-reperfusion injury (IRI) by suppressing neutrophil infiltration-associated oxidative stress [187]. This robust activity is attributed to the higher level of manganese-containing superoxide dismutase (MnSOD) in hUC-MSC-EVs compared to EVs derived from bone marrow (BM)-derived MSCs (BM-MSCs). Of note, this effect was nullified upon using siRNA targeting MnSOD [187].

Moreover, exosomes derived from human chemically derived hepatic stem cells (EXO-hCdH) have been shown to markedly attenuate the oxidative stress reactions and delay hepatocyte apoptosis [212]. EXO-hCdHs exerted protective effects against oxidative stress-mediated hepatocyte death by significantly activating nuclear factor erythroid 2-related factor 2 (NRF2) and its downstream target glutamate cysteine ligase (GCL), thereby modulating ROS generation.

#### Heart diseases

Numerous investigations have underscored the role of high oxidative stress in endothelial and vascular dysfunction can result from high oxidative stress, highlighting the importance of mitigating oxidative stress as a therapeutic approach for cardiovascular diseases in the elderly, with the potential to improve vascular function [213].

SOD-enriched human cardiac resident mesenchymal progenitor cells (CPCs-exos) exhibit potent ROS-reducing effects in rat ventricular myocytes exposed to trastuzumab or doxorubicin following i.v. administration [214]. This activity is attributed to miR-146a-5p encapsulated CPCs-exos, which suppress the doxorubicin-activated genes, namely NAD(P)H oxidase, myeloperoxidase (Mpo), interleukin-1 receptor-associated kinase (Irak1), tumor necrosis factor receptor-associated factor 6 (Traf6), Nox4, and signaling effector mothers against decapentaplegic protein 4 (Smad4). Additionally, neural progenitor cell-derived EVs (NPC-EVs) effectively protect endothelial cells (ECs) from angiotensin II-induced ROS production and subsequent apoptotic changes by delivering miR-210, which modulates VEGF/VEGFR2 and Nox2/ROS signaling pathways [215].

In acute myocardial infarction (AMI) in mouse cardiomyocytes, miR-23a-3p-enriched hUC-MSC-Exos inhibit the expression of divalent metal transporter 1 (DMT1), elevating GSH levels while reducing ROS and malondialdehyde (MDA) production [216]. Moreover, treatment with hUC-MSC-Exos decreases ROS production and ferroptosis in in vitro H/R-induced myocardial cells. Similarly, miR-214 abundant in BM-MSC-Exos suppresses calcium/calmodulin-dependent protein kinase II (CaMKΠ) expression in H2O2-treated cardiac stem cells (CSCs), leading to increased SOD levels and decreased ROS and MDA generation [217]. Interestingly, hypoxia-cultured MSCs exhibit greater enrichment of miR-214 and exert better therapeutic effects than those cultured under normoxic conditions. Intramyocardial injection of iPS-Exos before reperfusion in a mouse ischemic myocardium prevents myocardial ischemia/reperfusion (MIR) damage, attributed to the delivery of cardioprotective miRNAs such as HIF-1α-regulated miR-210 and Nanog-modulated miR-21 to H9C2 cells via iPS-Exo [218].

Furthermore, exosomes derived from H_2_O_2_-treated BM-MSCs (H-Exo) show high enrichment of miR-21 compared to untreated cells (N-Exo) and effectively suppress PTEN expression in C-kit^+^ cardiac stem cells (CSCs), activating the PI3K/AKT signaling pathway and protecting against oxidative stress-induced cell death [219]. Thus, MSC-Exos represents a promising therapeutic delivery system for C-kit^+^CSC treatments in the ischemic myocardium.

#### Musculoskeletal disorders

**Fig. 3:**
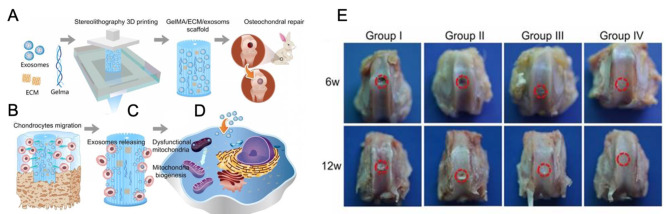
Fabrication, mechanism, and in vivo activity of 3D-printed cartilage extracellular matrix (ECM)/gelatin methacrylate (GelMA)/exosome scaffold in osteochondral defect rabbit model. (**A**) Osteochondral defect implantation with stereolithography-assisted ECM/GelMA/exosome bioprinting. (**B**) Chondrocytes migration to the defected areas. (**C**) Stereolithography-based 3D scaffold controlled the release of exosomes. (**D**) 3D scaffold/exosomes bioprinting boosts the mitochondrial biogenesis process in aberrant mitochondria. (**E**) Visual assessment of the healing process of the osteochondral defect areas at 6 and 12 weeks. This figure is reproduced from [222] after permission. This is an open-access article distributed under the terms of the Creative Commons Attribution (CC BY-NC) license (https://creativecommons.org/licenses/by-nc/4.0/). See http://ivyspring.com/terms for full terms and conditions

OA is characterized by oxidative stress damage and mitochondrial dysfunction, with a higher prevalence of mtDNA damage in OA patient chondrocytes compared to healthy counterparts [220, 221]. Efficient treatment of early OA has been demonstrated through the delivery of MSC-Exo using the 3D-printed cartilage ECM/GelMA/exosome scaffold (Fig. 3) [222]. This effect is attributed to their potential to restore oxidative stress-mediated chondrocyte mitochondrial dysfunction, promoting chondrocyte migration, and the polarizing of the synovial macrophage toward an M2 phenotype.

**Fig. 4:**
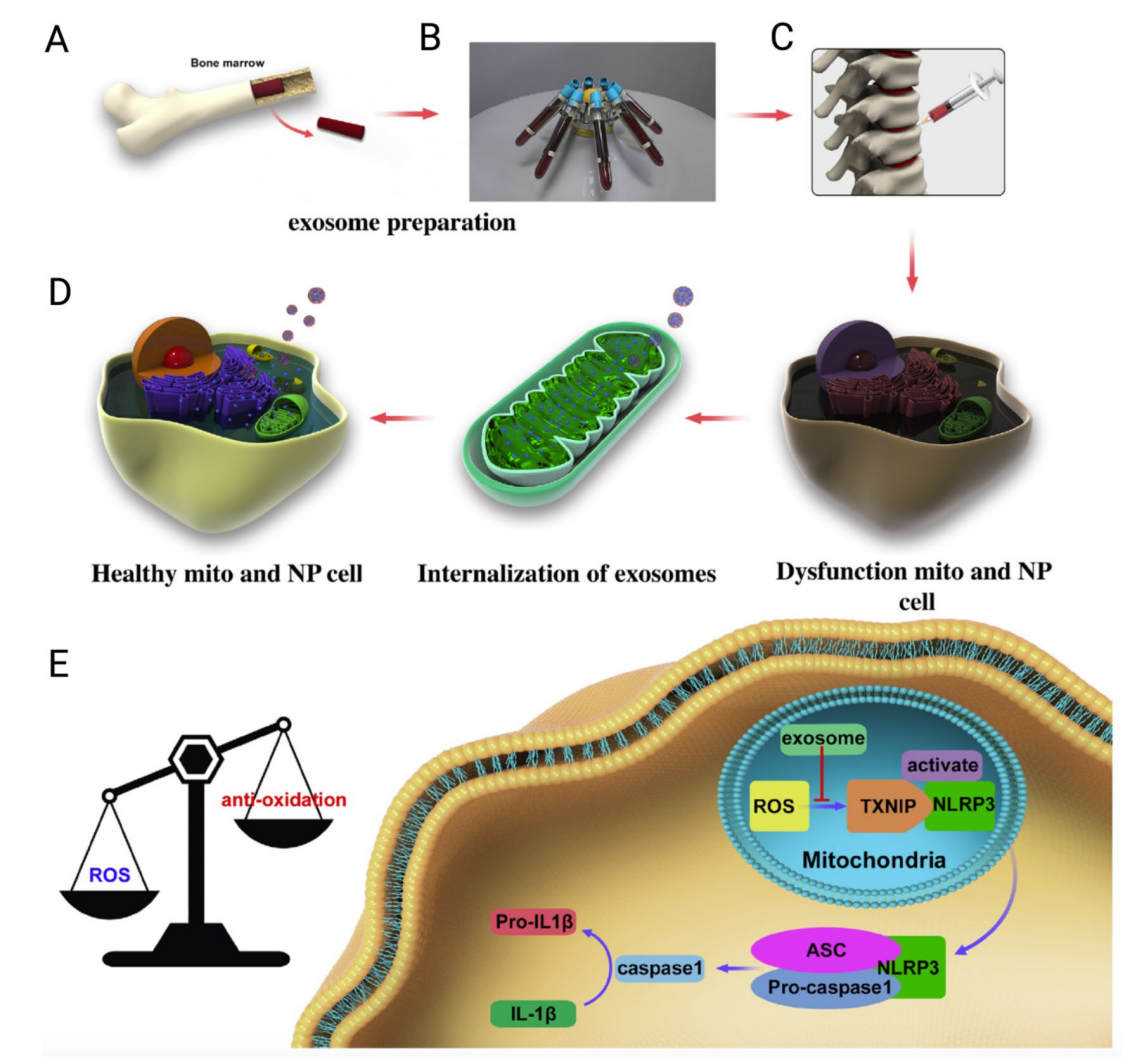
Representative diagram depicting isolation and the mechanism of BM-MSC-Exos in intervertebral disc degeneration (IVDD) therapy. (**A**) C57BL/6 mice-derived BM-MSCs are the source of BM-MSC-Exos. (**B**) Exosomes isolation via the centrifugation of MSC conditioned media. (**C**) Assessment of the therapeutic capacity of BM-MSC-Exos via their injection in IVDD rabbit model. (**D**) BM-MSC-Exos internalization and their role in promoting mitochondrial biogenesis. (**E**) The antioxidant activity of BM-MSC-Exo is ascribed to enhancing mitochondrial functions and inhibiting the NACHT, LRR, and PYD domain-containing protein 3 (NLRP3) inflammasome. This figure is reproduced from [46] with permission

Previous research has demonstrated a significant association between oxidative stress and the pathological progression of intervertebral disc degeneration (IVDD) [223]. A research study highlighted the potent in vitro and in vivo therapeutic efficacy of BM-MSC-Exos in the treatment of IVDD in vitro model using H_2_O_2_-induced nucleus pulposus (NP) and rabbit IVDD models, respectively [46]. This beneficial effect is attributed to the antioxidant properties of BM-MSC-Exo, which are implicated in enhancing mitochondrial function while concurrently suppressing the activity of the NACHT, LRR, and PYD domain-containing protein 3 (NLRP3) inflammasome (Fig. 4).

The hallmarks of cartilage degradation in OA prominently feature mitochondrial dysfunction and oxidative stress-associated damage [220]. Notably, intra-articular injection of primary chondrocyte-derived EVs in mice significantly attenuated OA progression, a phenomenon mediated by the restoration of the mitochondrial dysfunction and macrophage polarization towards the M2 phenotype. Both mitochondrial dysfunction and inflammation play pivotal roles in exacerbating the pathogenesis of OA, leading to cell death and matrix degradation [224, 225].

AD-MSC-Exo potently mitigated the in vitro OA model in chondrocytes stimulated with interleukin (IL)-1β, resulting in a significant reduction in the level of the inflammatory factors including tumor necrosis factor-α (TNF- α), NO, IL-6, and PGE_2_ [39]. In addition, AD-MSC-exo treatment to IL-1β-stimulated chondrocytes showed a decrease in the expression level of iNOS and a significant reduction in the nitrite level in the culture medium compared to untreated exosomes. Moreover, like MSC transplantation, BM-MSC-Exo mitigated radiation-induced bone loss in rats. Comparative analysis between BM-MSCs treated with exosomes and those receiving irradiation alone revealed that exosome treatment markedly reduced oxidative stress, facilitated DNA damage repair, decreased growth inhibition, and lowered expression of cell senescence-associated proteins [226]. Mechanistically, BM-MSC-exo stimulated β-catenin expression in BM-MSCs post-irradiation, thereby restoring the balance between osteogenic and adipogenic differentiation.

#### Renal disorders

A research group isolated exosomes from melatonin-treated healthy MSCs (MT exosomes) and investigated the biological functions of MT exosome-treated MSCs isolated from chronic kidney disease (CKD) patients (CKD-MSCs) in improving therapeutic potential in CKD patients [130]. Evaluation of MT exosome-treated MSCs isolated from CKD patients (CKD-MSCs) revealed an increased expression of miR-4516, which is involved in the melatonin-mediated upregulation of cellular prion protein (PrPC) in MT exosomes. This enhancement resulted in significant improvements in the proliferation, senescence, and mitochondrial function of CKD-MSCs upon treatment with MT exosomes. Furthermore, CKD-MSCs treated with MT exosomes demonstrated enhanced functional recovery and vascular repair in a mouse hindlimb ischemia model with chronic kidney disease. Previous reports have indicated that the PrPc in exosomes enhances the immunomodulatory action and increases the expression of antioxidant proteins in the cell [227, 228], thereby augmenting MSCs regrowth capacity and reducing oxidative stress caused by ischemia.

Furthermore, a study highlighted the robust antioxidant activity of hWJMSC-derived MVs (hWJMSC-MVs) in ameliorating renal ischemia/reperfusion injury (IRI) was demonstrated in vitro and in vivo [229]. Hypoxia-induced injury in HUVEC and NRK-52E cell lines is efficiently recovered upon hWJMSC-MVs treatment. Moreover, the i.v. injection of hWJMSC-MVs in rats subjected to unilateral kidney ischemia showed a recovery of renal fibrosis and restoration of renal function after two weeks of injection. This effect is attributed to the downregulation of the NOX2 expression levels.

In 2022, Nguyen et al. employed a model of acute renal injury using H_2_O_2_-exposed renal tubuloids on cell culture inserts in the chip and then tested the effect of BM-MSC-Exo on amelioration of oxidative damage-associated kidney injury [230]. Interestingly, treatment of BM-MSC-Exo into the circulating medium potently restored the functional integrity of the kidney epithelial barrier and improved the transport function of the renal tubules.

Using a SIRT1-deficient unilateral ureteral obstruction (UUO) mouse model, a research report unveiled grave renal apoptosis and fibrosis as well as increased vulnerability to oxidative stress in comparison to the wild type [231]. Using a lentiviral transfection method, glial cell line-derived neurotrophic factor (GDNF) was transfected into hAD-SCs, and exosomes (GDNF-AMSC-Exos) were isolated [232]. GDNF-AMSC-Exos are effective in improving renal fibrosis and peritubular capillary (PTC) rarefaction in the UUO mouse model. The mechanism responsible for mitigating PTC loss and exerting strong protective effects seemed to enhance SIRT1 expression in the kidney, along with the upregulation of phosphorylated endothelial nitric oxide synthase (p-eNOS). Moreover, in vitro, GDNF-AMSC-exos improved endothelial cell migration and angiogenic activity while decreasing apoptosis. SIRT1 was essential for the GDNF-AMSC-Exo-dependent protective action on HUVEC against hypoxia and serum deprivation damage via boosting endothelial angiogenesis, and this effect is significantly decreased by silencing SIRT1.

An investigation demonstrated that hucMSC-Exo can reverse cisplatin-induced acute kidney injury (AKI) in rats and rat renal tubular epithelial (NRK-52E) cell damage by reducing oxidative stress and cell death and enhancing cell proliferation [60]. hucMSC-Exo can counteract the cisplatin-mediated oxidative stress by diminishing the production of toxic chemicals (8-OHdG, MDA), elevating GSH levels, inhibiting the p38MAPK pathway, and activating ERK1/2 signaling.

#### Skin diseases

**Fig. 5:**
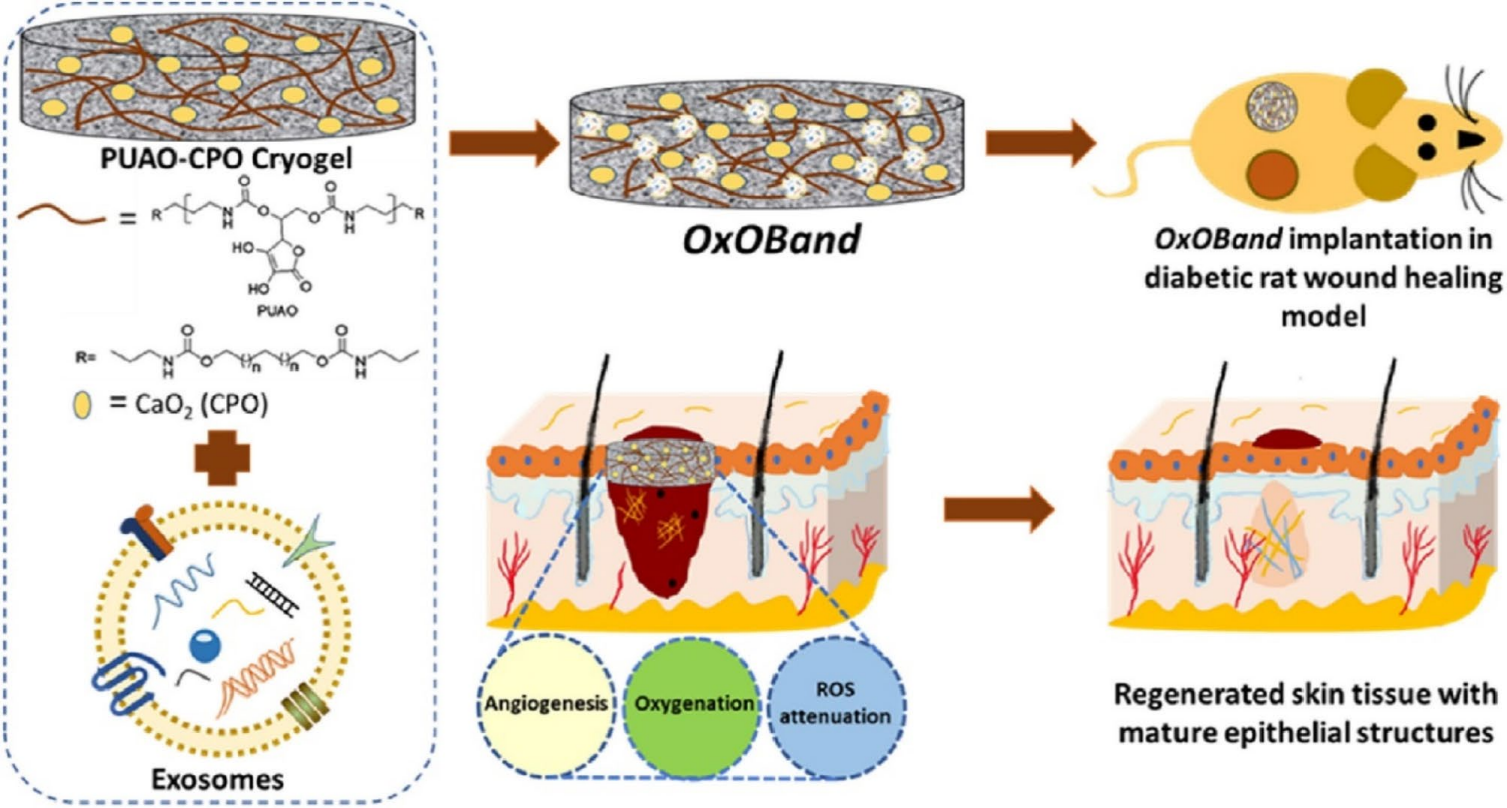
Schematic illustration of the conjugation of AD-SC-Exo and OxOBand scaffolds and their therapeutic activity against the infected wound. AD-SC-Exo/OxOBand boosts the angiogenesis and collagen deposition. This figure was reproduced from [42] with permission

Research reports have highlighted the synergistic effect of AD-SC-Exoin combination with the oxygen-releasing antioxidant wound dressing OxOBand scaffold [42]. OxOBand comprises antioxidant polyurethane (PUAO) integrated with AD-SC-Exo, resulting in highly porous cryogels with prolonged oxygen release capabilities. This combination of the scaffold and AD-SC-Exo demonstrated potent enhancement in the regeneration of diabetic wound ulcers infected with *S. aureus*, and *P. aeruginosa* by reducing oxidative stress, boosting re-epithelization, facilitating collagen deposition, increasing angiogenesis, and upregulating the expression level of VEGF and CD31 (Fig. 5).

One research study showcased the potent protective effects of MSC-Exos both in vitro and in vivo, using H_2_O_2_-treated keratinocytes, and in vivo, with ultraviolet (UV)-exposed mouse skin [233]. This beneficial impact is attributed to the robust antioxidant activity of MSC-Exo, which facilitates the inhibition of oxidative stress-associated DNA damage and mitochondrial abnormalities. Notably, Nrf2 knockdown abrogates the MSC-Exo antioxidant mechanism in skin injury repair, underscoring the involvement of the Nrf2 defense mechanism in the antioxidant activity of MSC-Exo.

#### Ocular, digestive, and reproductive diseases

The antioxidant potential of adipose-derived stem cells (ASC-Exos) was demonstrated when co-cultured with UVB-pretreated human lens epithelial cells (HLECs), resulting in the prevention of apoptosis, reduction in Ca^2+^ level, and downregulation of cartilage acid protein 1 (CRTAC1) expression, mediated by miR10a-5p [234]. This study sheds light on the implications and underlying mechanisms of antioxidant EVs in cataract therapy.

In a premature ovarian insufficiency (POI) mice model and primary granulosa cells (hGCs) isolated from POI patients, human amniotic MSC-derived exosomes (hAMSC-Exos) rich in miR-320a effectively mitigated ROS levels [235]. This effect is mediated by downregulating sirtuin 4 (SIRT4), leading to decreased expression of downstream target genes including AMP-dependent kinase, adenine nucleotide translocator 2, and GTPase optic atrophy type 1.

**Fig. 6:**
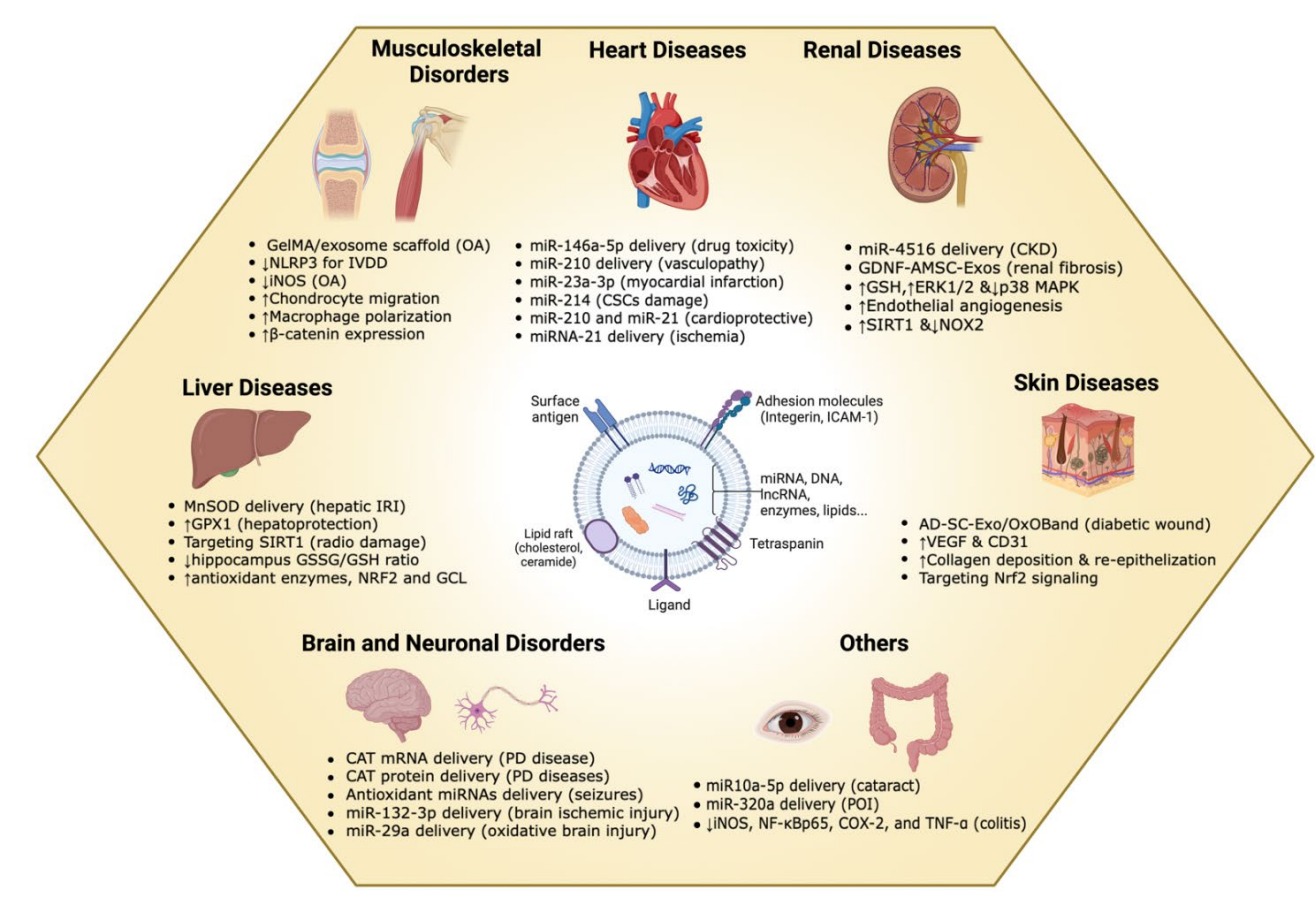
Schematic diagram summarizing the role of the ROS-scavenging capacity of EVs in tissue regeneration with the possible mechanisms. EV’s ROS scavenging capacity is involved in the regeneration of several tissue disorders including brain, heart, liver, kidney, skin, musculoskeletal, and ocular disorders. This robust activity is attributed to the delivery of therapeutic miRNAs, specific antioxidant proteins, and modulating key signaling pathways. ↑:upregulation; ↓:downregulation. This figure was created with BioRender.com, accessed on December 23rd, 2023

The antioxidant capacity of BM-MSC-EVs played a pivotal role in the ameliorating 2,4,6-trinitrobenzene sulfonic acid (TNBS)-induced colitis in rats following i.v. injection [236]. This potent effect is characterized by histological restoration, decreased gene and protein expression of inducible nitric oxide synthase (iNOS), nuclear factor kappaBp65 (NF-κBp65), cyclooxygenase-2 (COX-2), and TNF-α in injured colon tissue. Moreover, BMSC-EVs administration reduced interleukin-1β (IL-1β) expression levels while increasing interleukin-10 (IL-10) expression. Elevated levels of SOD and glutathione (GSH), alongside decreased MPO activity and MDA levels, indicated the attenuation of oxidative disturbances. The ROS-scavenging capacity of EVs implicated in tissue regeneration is summarized in Table 3; Fig. 6.

**Table 3:** EV-mediated ROS modulation for tissue regeneration

Exosome source	Cargo/Content	In vitro/in vivo application	Target disease	Effects/Mechanism	References
EXOtic devices aiding the efficient customizable generation of designer exosomes	CAT mRNA delivery	Subcutaneous transplantation in 6-OHDA-injected mice	PD disease	Mitigating of neurotoxicity and neuroinflammation in vitro and in vivo.	[41]
Ex vivo EVs loading with CAT protein	CAT protein delivery	-Increasing In vitro accumulation of exoCAT in the PC12 neuronal cells -Mitigating ROS levels in LPS-activated macrophages &↑delivery in PD mouse brain (Intranasal injection)	PD disease	Alleviating neuroinflammation in 6-OHDA-induced mouse model	[199]
hUC-MSC-Exo-enriched in antioxidant miRNAs	Antioxidant miRNAs delivery: miR-215-5p, miR-424-5p, miR-31-3p, miR-193b-3p, and miR-200b-3p	H_2_0_2_-treated hippocampal neurons and pilocarpine-induced seizures mouse model	Seizures	Targeting Nrf2 signaling pathway	[188]
mASC-Exos	n/a	Motoneuron-like NSC-34 cells expressing ALS mutations and H_2_0_2_ treatment	fALS	Neuroprotective activity	[200]
BM-MSC-Exos	miRNA delivery (miR-132-3p)	-H/R-injured ECs (In vitro) - Focal ischemic stroke mouse model (i/v injection)	Brain ischemic injury	-Reducing ROS generation (H/R-injured ECs) and enhancing PI3K/AKT/eNOS signaling	[201]
miR-29a-enriched astrocyte-Exo	miRNA delivery	-Mouse microglia N9(oxygen and glucose deprivation in vitro model) -BIRI rat model	Brain injury (oxidative stress-dependent)	-Reducing MDA, cell death, and inflammatory cytokines, Increasing SOD and reducing TP53INP1 expression -Targeting NF-κB/NLRP3 pathway	[202]
AFSC-Exos	n/a	Primary neuron culture from AD mice	AD disease	-Increasing antioxidant enzymes TrxR1, TrxR2, GSH, and SOD1, Increasing PI3K/AKT signaling, and reducing NOX4 activity	[203]
BM-MSC-Exos	Endogenous CAT secretion	Rat hippocampal neurons	AD disease	Neuroprotective function and preventing synapse damage	[186]
AD-MSC-Exos	n/a	Intranasal administration in rats with chronic alcohol consumption condition	Chronic alcohol intake	-Reducing chronic alcohol intake by 84% -Reducing hippocampus GSSG/GSH ratio, increasing GLT1 expression, and reducing inflammatory microglia	[45]
AD-MSC-Exos	n/a	Irradiation-exposed rat microglia	Radiation-associated oxidative damage	-Mitigating inflammation and oxidative stress & decreasing CD68 expression in microglia -Targeting SIRT1 pathway	[204]
hUC-MSC-Exo	n/a	-CCl_4_ exposed mouse -H_2_O_2_-treated L02 Liver Cells	Hepatic failure	GPX1-mediated hepatoprotective activity	[40]
hUC-MSC-EVs	MnSOD delivery	-IRI mouse model -H_2_O_2_-treated L02 liver cells	Hepatic IRI	Reducing neutrophil infiltration and apoptosis	[187]
Human chemically derived hepatic stem cells	n/a	-H_2_O_2_-treated hepatocytes	Hepatic cell toxicity	-Decreasing cell death (inhibiting PARP cleavage), increasing the expression of antioxidant enzymes, NRF2 and GCL.	[212]
CPCs-Exos	miRNA delivery: miR-146a-5p	-Dox-treated myocytes (In vitro model) -Dox-injected rat model	Oxidative stress-mediated cardiomyocyte toxicity	-Decreasing expression of Dox-associated genes: Irak1, Traf6, Mpo, Nox4, and Smad4 -In vivo activity: alleviating myocardial fibrosis, decreasing iNOS expression, decreasing macrophage infiltration, and decreasing LV dysfunction	[214]
NPC-EVs	miR-210 delivery	Ang II-exposed EC	Vasculopathy	Targeting VEGF/VEGFR2 and Nox2/ROS signaling pathways	[215]
hUC-MSC-Exos	miR-23a-3p delivery	-AMI mouse model (In vivo) -H/R-induced myocardial cell ferroptosis (In vitro)	Myocardial infarction	-Decreasing DMT1, increasing GSH LEVEL, decreasing Ferroptosis, and decreasing iron deposition	[216]
BM-MSC-Exos	miR-214 delivery	H_2_O_2_-treated CSCs	Oxidative stress-mediated CSCs damage	-Inhibiting apoptosis, decreasing CaMKΠ, and increasing SOD level	[217]
iPS-Exos	Cardioprotective miRNAs delivery (miR-210 and miR-21)	-H_2_O_2_-treated H9C2 cells -MIR mice model (intramyocardial injection)	Myocardial ischemia	-Decreasing Caspase 3/7 activation	[218]
BM-MSC-Exo	miR-21 delivery	H_2_O_2_-treated C-kit^+^CSCs and MSCs	Ischemic myocardium	Decreasing PTEN expression and enhancing PI3K/AKT signaling	[219]
GelMA/MSC-Exo scaffold	n/a	-Rotenone-treated chondrocytes -Osteochondral defect rabbit models	OA	Promoting chondrocyte migration, and macrophage polarization	[222]
BM-MSC-Exo	n/a	-H_2_O_2_- NP (In vitro) and Rabbit IVDD model	IVDD	Boosting Mitochondrial function and suppressing NLRP inflammasome	[47]
AD-MSC-Exo	n/a	IL-stimulated chondrocytes	OA	-Decreasing TNF- α, IL-6, NO, and PGE_2_ and decreasing iNOS, and nitrite levels in the medium	[39]
BM-MSC-Exo	n/a	-Radiation-exposed BM-MSCs (In vitro) -Radiation-mediated bone loss in rats (In vivo)	Radiation-mediated bone loss	-Increasing β-catenin expression and recovery of the osteogenic/adipogenic differentiation equilibrium	[226]
Melatonin-treated healthy MSCs (MT exosomes)	miR-4516 delivery	-CKD-MSCs -Mouse hindlimb ischemia model	CKD	-Promoting proliferation and the mitochondrial function of CDK-MSCs, increasing PrPC in MT-Exo, and In vivo vascular recovery	[130]
BM-MSC-Exo	n/a	H_2_O_2_-treated renal tubuloids	Acute renal injury	Enhancing restoration of barrier integrity and rescue of functional transport	[230]
GDNF-AMSC-Exos	n/a	HUVEC hypoxia/serum deprivation damage and UUO mouse model	Renal fibrosis	-Promoting endothelial angiogenesis, increasing SIRT1 expression, and improving renal fibrosis in vivo	[232]
hucMSC-Exo	n/a	-Cisplatin-treated NRK-52E cells and AKI rat model	Nephrotoxicity	-Suppressing p38 MAPK pathway, activating ERK1/2 signaling, increasing GSH level	[61]
hWJMSC-MVs	n/a	-Hypoxia-exposed HUVEC and NRK-52E cells (In vitro) and IRI rat model (i.v. injection)	Renal failure	Decreasing fibrosis and renal function restoration and downregulating NOX2 expression	[229]
AD-SC-Exo/ OxOBand scaffold	n/a	-HaCaT cell in vitro migration	Infected diabetic wound ulcers	Promoting re-epithelialization, enhancing angiogenesis, increasing collagen deposition, and increasing VEGF and CD31	[42]
MSC-Exo	n/a	-H_2_O_2_-treated keratinocytes and UV-exposed mice skin	Radiation-mediated skin damage	Alleviating DNA damage and mitochondrial deformities and Nrf2-dependent mechanism	[233]
ASC-Exos	miR10a-5p delivery	UVB-exposed HLECs	Cataract	-Decreasing CRTAC1 expression and decreasing apoptosis and Ca^2+^ level	[234]
hAMSC-Exos	miR-320a delivery	hGCs from POI patients	POI	-Downregulating SIRT4 and downstream genes	[235]
BM-MSC-EVs	n/a	TNBS-induced colitis rat model (i.v. injection)	Colitis	-Decreasing iNOS, NF-κBp65, COX-2, and TNF-α and increasing SOD and GSH	[236]

*Abbreviations* ROS, reactive oxygen species; CAT, catalase; AD, Alzheimer’s disease; PD, Parkinson’s disease; fALS, familial amyotrophic lateral sclerosis; H_2_O_2_, hydrogen peroxide; SOD, superoxide dismutase; hUC-MSC, human-umbilical cord mesenchymal stem cell; OA, osteoarthritis; 6-OHDA, 6-hydroxydopamine; Nrf2, nuclear factor-erythroid 2-related factor 2; H/R-injured ECs, hypoxia/reoxygenation-injured cerebral endothelial cells; BIRI, brain ischemia-reperfusion injury; TrxR1, thioredoxin reductase 1; GLT1, glutamate transporter 1; Hepatic IRI, hepatic ischemia-reperfusion injury; DOX, doxorubicin; LV, left ventricular; iNOS, inducible nitric oxide synthase; DMT1, divalent metal transporter 1; GelMA, methacrylated gelatin; NLRP, nitrogen lipid regulator protein; SIRT1, sirtuin1; HUVEC, human umbilical vein endothelial cell; UVB, ultra violet B; TNBS, trinitrobenzene sulfonic acid; NF-kBp65, nuclear factor-kappa B p65; CRTAC1, cartilage acid protein 1; i.v., intravenous; n/a, not applicable

## Application of engineered EVs in tissue regeneration

Within the realm of tissue repair and rejuvenation, EVs have emerged as a groundbreaking tool, introducing unprecedented precision to therapeutic interventions [225, 237]we need to add this reference here:Chun, C., Smith, A. S., Kim, H., Kamenz, D. S., Lee, J. H., Lee, J. B., ... & Kim, D. H. (2021). Astrocyte-derived extracellular vesicles enhance the survival and electrophysiological function of human cortical neurons in vitro. Biomaterials, 271, 120700. Traditionally, regenerative approaches have relied on cell-based therapies, tissue grafts, and biomaterial scaffolds, each with its distinct advantages and limitations [48, 238, 239]. The advent of engineered EVs has revolutionized this field by harnessing the innate regenerative capabilities of these nanoscale messengers while also utilizing their cargo and targeting properties [240, 241].

In this section, we delve into the intricate designs and mechanisms of engineered EVs that underlie their therapeutic potential for various diseases. We will navigate the promising field of regenerative medicine, with a focus on tissue-specific regeneration, including bone, skeletal muscle, and peripheral nerves. These are the most investigated tissues with the application of engineered EVs. From conditions like fractures, osteoporosis, and OA impacting bone health to muscular dystrophies, sports injuries, age-related muscle wasting, and a range of peripheral nerve system (PNS) injuries and disorders, there exists a pressing need for innovative and effective therapeutic strategies [143, 144]. Engineered EVs offer a promising avenue in the pursuit of more precise and regenerative treatments for these debilitating disorders.

### Bone regeneration

EVs hold promise for enhancing intracellular signaling in target cells and delivering cargo components to trigger osteoblast proliferation and differentiation [242]. However, the therapeutic application of natural EVs for bone repair faces constraints [90], underscoring the urgent need for engineered EVs to enhance their therapeutic efficacy.

MSC-Exos exhibit a robust therapeutic effect on bone abnormalities by targeting bone tissues and inducing osteogenic differentiation, thereby accelerating bone repair processes [24, 243]. In osteoporotic rat model, EVs derived from human induced pluripotent stem cells (hiPSC) have been shown to mediate bone regeneration [244]. Similarly, in the rabbit model of femoral head osteonecrosis, EVs isolated from BM-derived stem cells (BMSCs) enhance osteoblast proliferation and osteogenic differentiation, thereby promoting osteogenesis [245].

EVs directly contribute to bone repair by delivering cargos that modulate specific downstream signaling pathways within cells [246, 247]. Various miRNAs present in MSC-EVs, such as miRNA-135b, miRNA-204, and miRNA-196a, play significant roles in controlling bone regeneration process [248–250]. MSC-Exos activate BMP/Smad, Wnt/-catenin, and PI3K/AKT signaling pathways, thereby promoting osteoblast growth, proliferation, and recruitment of endogenous MSCs to bone defective sites [251–253]. Moreover, EVs activate local angiogenesis, inhibit bone resorption, and activate the AKT/mTOR signaling pathway, further enhancing osteogenesis [254, 255].

Additionally, the immunomodulatory function of EVs plays a crucial role in suppressing osteoclast activity and promoting vasculature and osteogenesis [251]. In a complex environment, MSC EVs influence immune cell activity [243, 256]. For instance, MSC-EVs drive the polarization of macrophages from the M1 to the M2 phenotype by delivering intracellular cargo, contributing to the establishment of an anti-inflammatory milieu during bone defect regeneration [257].

**Fig. 7:**
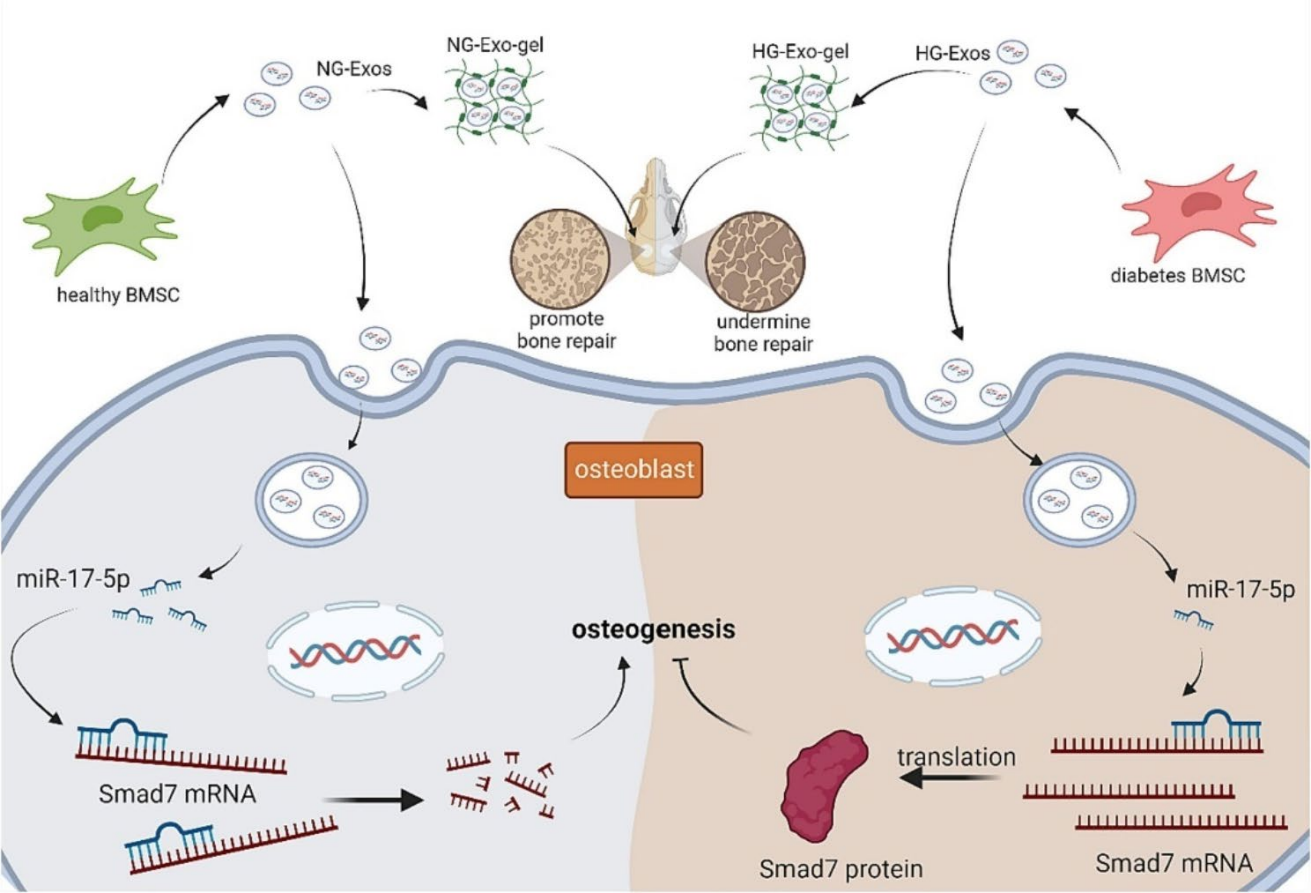
Schematic diagram illustrating the comprehensive mechanisms of high glucose (HG)- and normal glucose (NG)-Exos in the regeneration of bone defects and osteogenesis of rat osteoblasts (rOBs). Under non-diabetic conditions, miR-17-enriched NG-Exos is rich in miR-17, which stimulates osteogenic differentiation of rOBs and bone repair in T2DM rats via targeting Smad7. Under HG culture (diabetic condition), HG-Exos have low miR-17, which leads to an upregulation of SMAD7 expression and, as a result, an impediment to rOB osteogenesis and rat bone regeneration. This figure is reproduced from [259] after permission

Diabetes impairs dental regrowth by interfering with bone metabolism, increasing the risk of periodontitis due to hyperglycemia and the constant production of inflammatory factors [258]. Diabetes mellitus (DM)-related complications, such as diabetic bone disease (DBD) are defined by decreased osteocyte activity and delayed bone remodeling as a result of elevated blood glucose levels and prolonged inflammatory factor production. HG-Exos, exosomes prepared from BM-MSCs cultured in high-glucose (HG) medium to emulate diabetic conditions, impede the in vitro migration and osteogenesis of rat osteoblasts (rOBs), and bone regeneration in rats in vivo [259]. In contrast, NG-Exos (isolated from BM-MSCs cultured in normal-glucose medium (NG) promotes osteogenesis and migration of rOBs in vitro and bone regeneration in T2DM rats in vivo. Reduced expression l of miR-17-5p in the skulls of rats with T2DM, HG-Exos, and HG-Exo–co-cultured rOBs. This subsequently suppresses SMAD7 expression and contributes to inadequate bone repair (Fig. 7).

MSCs pre-stimulated with 20 ng of TNFα/mL for 72 h produced EVs that exhibited enhanced efficacy in polarizing primary mouse macrophages from the pro-inflammatory M1 to the regenerative M2 phenotype [260]. Surprisingly, in contrast to cultures not preconditioned with TNFα, the cargos of pre-stimulated MSC-EV were enriched in anti-inflammatory miRNAs, including miR-15b, miR-19b, and miR-22. Consequently, these EVs demonstrated greater regenerative potential when applied to a rat calvarial injury model, leading to increased bone production alongside reduced inflammation [260]. Similarly, it has been reported that pre-stimulation of MSC with parathyroid hormone (PTH) 1–34 could enhance the MSC-EV curative effect against OA [261]. In this study, BMSC were treated with PTC for 6 h, followed by media change and incubation for 48 h before EV isolation. Results on the in vitro model of OA in chondrocytes demonstrated that EVs isolated from PTH-pretreated MSCs potently mitigated the expression levels of inflammatory factors TNF-α, IL-2, and IL-6, while significantly boosting proliferation, migration, and extracellular matrix production more significantly than EVs derived from control unstimulated MSCs [261].

Increased inflammation has been associated with osteoporosis in diabetics [262]. In such cases, the anti-inflammatory properties of MSC-EV may be beneficial in inhibiting or treating disturbances to bone metabolism in diabetics. Compared to untreated rats, an i.v. administration of AD-MSC-Exos markedly prevented the decrease in bone mineral density in rats with streptozotocin-induced diabetes and osteoporotic bone loss. This effect was accompanied by a reduction in inflammatory cytokine release following the suppression of NLRP3 inflammasome activation in osteoclasts [263].

Vascularization is crucial for supplying nutrients and growth factors and for controlling bone production and regeneration following a bone injury or fracture. Bone development initiates in close association with the physical architecture of blood vessels and vascular cells [264]. Notably, EV release play a role in promoting osteogenesis and angiogenesis, processes supported by communication among different cell types in bone tissue, including endothelial cells, osteoclasts, osteoblasts, and stem cells [265]. VEGF is essential for stimulating angiogenesis and bone regeneration by hMSC-EVs. In a rat model of calvarial bone defects, it was demonstrated that co-administration of anti-VEGF antibodies with hMSC-EVs hindered the beneficial effects of EVs on bone regeneration [266].

MSC-EVs contain various RNA classes that promote angiogenesis, including long noncoding RNA-H19 (lncRNA-H19). The i.v. administration of lncRNA-H19-loaded EV-BMSC enhanced angiopoietin 1 (ANGPT1) secretion, promoted angiogenesis, and rescued bone loss in an osteoporotic mouse model [267]. This effect was attributed to the modulation of the ANGPT1 gene by miR106a, which is involved in angiogenesis and stimulation of osteoblastogenesis [268].

A bioglass scaffold with GelMA/nanoclay hydrogel conjugated with miR-23a-3p-enriched UCMSC-Exos significantly enhanced in vitro osteogenesis and ameliorated cranial bone deformities in an in vivo animal model by targeting AKT signaling and inhibiting PTEN [269]. This effect was associated with boosted angiogenesis-mediated bone repair. Various mechanisms of MSC-EVs are implicated in bone regeneration, such as enhancing cell migration, EVs produced from hypoxia-exposed MSCs, and their role in enhancing the differentiation of bone cells, are extensively reviewed elsewhere [264, 270].

### Skeletal muscle repair

Skeletal muscle possesses an inherent ability to regenerate to a certain extent, primarily facilitated by the activation of satellite cells, specialized myogenic stem cells residing in the muscle tissue [271, 272]. However, in cases of severe injury or chronic degenerative conditions, this natural regenerative capacity may be compromised. Skeletal muscles are susceptible to a variety of disorders such as muscle dystrophy, atrophy, and inflammatory or metabolic myopathies [273, 274]. EVs offer a promising avenue to indirectly guide the repair of skeletal muscle tissue in many cases.Engineered EVs can be customized with specific cargos, including growth factors, microRNAs, or proteins, known to stimulate myogenic activity [275]. These bioactive payloads have the potential to promote satellite cell activation, muscle cell differentiation, and overall tissue repair [276–278]. For instance, previous studies have demonstrated that myomiRs like miR-206, miR-133a, and miR-1 play crucial roles in myoblast proliferation [279]. Additionally, in case of chronic inflammation followed by skeletal muscle injury, EV miR-223, the most abundant miRNA in peripheral blood, has been implicated in modulating the inflammatory response to skeletal muscle damage [280–283]. Studies have demonstrated that miR-223 down-regulates the expression of TNF-α and other pro-inflammatory molecules, inhibits inflammatory cell infiltration, and decreases the extent of necrotic muscle tissue [278, 284]. Consequently, miR-223 holds promise as a potential biomarker and therapeutic target for addressing chronic inflammation associated with inadequate muscle regeneration and repair following injury.

In addition to miRNA, peptides and proteins can also function as cargo in EVs. For example, a study investigated the engineering approach of inhibiting myostatin to improve the outcome of Duchenne muscular dystrophy (DMD) [285]. They demonstrated that a myostatin pro-peptide can be administered by conjugating its inhibitory domain into the extracellular loop of CD63, forming a complex called EXOpro. This complex, with the pro-peptide fused onto the surface of the exosome isolated from NIH3T3 fibroblasts, efficiently bound mature myostatin in serum, sequestering approximately 51% of circulatory myostatin after injection in mice. Notably, in mdx mice, EXOpro demonstrated a dose-dependent effect, significantly increasing muscle growth at higher doses [285].

Second, a key advantage of engineered EVs is their ability to be directed to specific cell types within the muscle tissue, a concept known as targeted delivery. Surface modifications, such as ligand-receptor interactions or antibody conjugation, enable precise homing to injured muscle cells, enhancing the therapeutic impact while minimizing off-target effects [286]. For instance, recent research has highlighted the potential of developing a myotropic drug delivery system within muscle tissue. In one study, EVs derived from human embryonic kidney (HEK) cells were engineered to display myotropic transmembrane proteins, including MyoMixer (MYMX), MyoMaker (MYMK), and M-Cadherin (M-CAD), on their surfaces. The incorporation of these proteins into EVs and their ability to deliver fluorescent-labeled cargo into mouse myotubes were assessed [287]. Among the candidates, MYMK demonstrated the highest propensity to incorporate into EVs and exhibited a significant increase in cargo delivery to myotubes. Additionally, the study investigated the endogenous protein cargo of non-engineered HEK293-EVs. These findings suggest the potential application of MYMK-EVs as a delivery platform for myotropic drugs, with further investigations needed to understand their in vivo effects and tissue tropism [287]. 

In addition to their direct effects on muscle cells, engineered EVs can modulate the immune response within the injured muscle [288], thereby regulating the clearance of cellular debris and facilitating a smoother regenerative process. Muscle injuries often trigger inflammatory responses that can exacerbate tissue damage. Engineered EVs can be tailored to contain those anti-inflammatory molecules, mitigating the detrimental effects of inflammation, and creating a more conducive environment for regeneration [289, 290]. One study revealed that myoblast releases EVs rich in miRNAs in inflammatory environments, transferring miR-224 to macrophages and inhibiting M2 polarization. Additionally, the study identifies WNT-9a as a potential target of miR-224 for macrophage polarization. In return, secretions from pro-inflammatory M1 macrophages hinder myogenic differentiation and promote cell proliferation [291].

Another research group isolated two types of EVs (EVNormo and EVHypo) from MSCs exposed to different oxygen conditions and found them to be efficiently taken up by BM-derived macrophages, promoting their shift from an inflammatory M1 to a pro-regenerative M2 phenotype. In an in vivo model of skeletal muscle damage, both types of EVs interacted with macrophages and led to reduced inflammation and enhanced markers of tissue repair, with EVHypo showing even more significant effects [185]. These findings both suggest that engineered MSC-derived EVs have robust anti-inflammatory properties, making them promising and practical therapeutic agents for skeletal muscle repair.

**Fig. 8:**
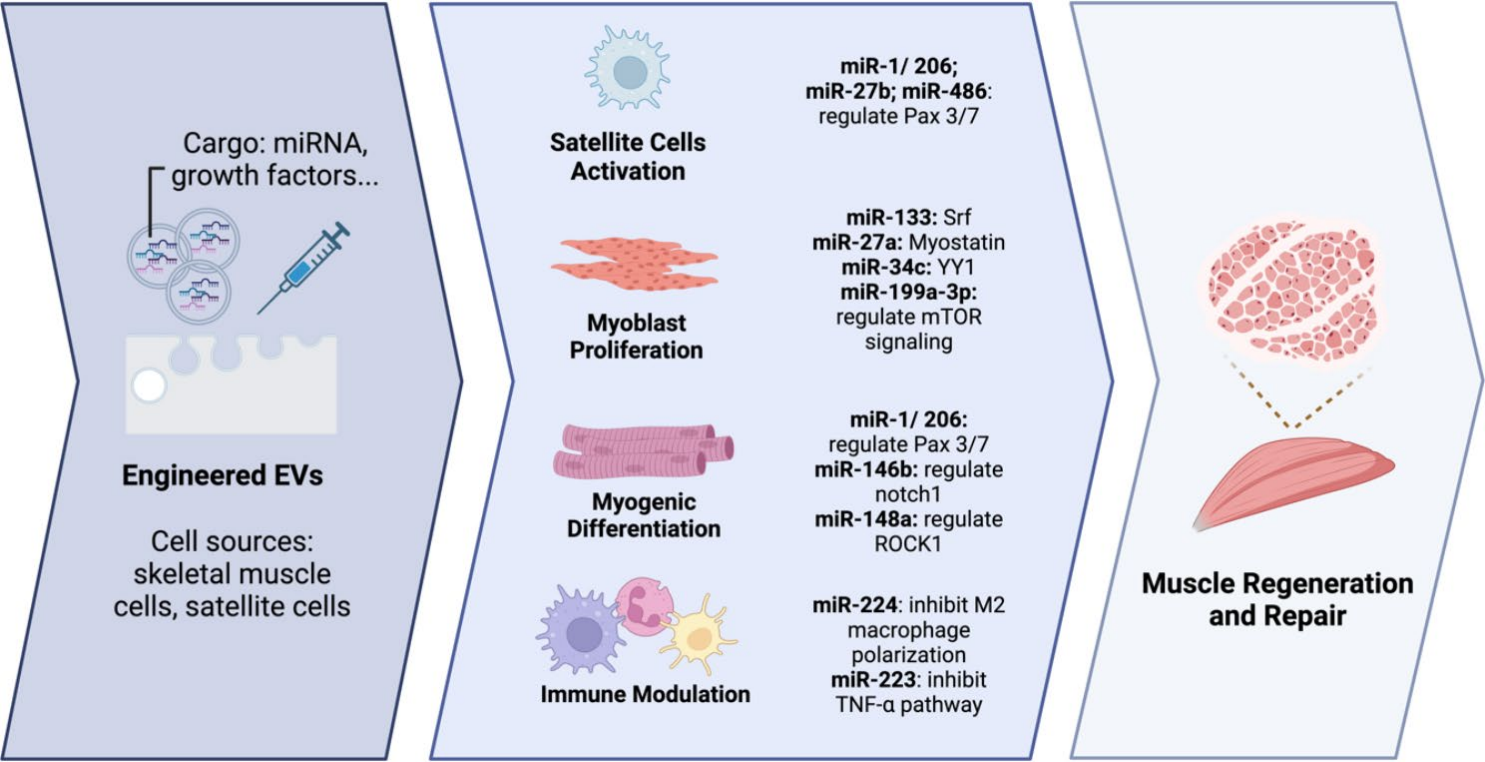
Schematic illustration of skeletal muscle regeneration with engineered EVs. The regulated gene or downstream mechanism of miRNA in each myogenic step is emphasized. This figure was created with BioRender.com, accessed on December 20th, 2023

In summary, Fig. 8 summarizes the myogenic steps and immune modulation principles behind skeletal muscle repair.

### Ameliorating peripheral neuropathy

Peripheral nerves are delicate yet vital structures, acting as the information highways that connect the central nervous system to every part of the body, enabling the brain to receive sensory input and command motor functions [292]. They play a crucial role in our ability to move, sense our surroundings, and react to the world around us. Damages or dysfunction of the peripheral nerves can result in sensory or motor deficits, pain, loss of coordination and balance, or muscle atrophy [293, 294]. Recent studies have shown promising results where engineered EVs are used to mitigate symptoms and reverse damage in peripheral neuropathy models. These EVs can be loaded with specific RNA molecules to modulate gene expression in target cells, paving the way for repairing or regenerating damaged nerve tissues.

The majority of the research about peripheral nerve disorders focuses on diabetic peripheral neuropathy (DPN). One study investigates the use of engineered MSC-Exo enriched with microRNA-146a for treating DPN. The exo-146a treatment accelerates improvements in nerve conduction velocity and sensory thresholds, demonstrating superior efficacy over naïve EVs [295]. This enhancement is partly due to the robust anti-inflammatory properties of miR-146a, which suppresses inflammation-related genes and pathways such as Toll-like receptor 4 (TLR4)/NF-κB signaling, leading to a reduction in inflammatory monocytes and endothelial activation. Additionally, the exo-146a treatment shows pronounced effects on neurovascular function, immunosuppression, and endothelial dysfunction attenuation, contributing to the overall therapeutic outcome in diabetic mice [295].

In addition to the naïve mesenchymal stromal cell derived EVs, one research team fused them with polypyrrole NP, aiming to regenerate nerves affected by DPN. They also applied electric stimulation for nerve repair and regeneration, providing guidance cues for myelination and axonal recovery post-injury. The study reported significant improvements in nerve functionality, measured by nerve conduction velocity and muscle action potential, in treated animals compared to controls, indicating effective nerve conductance restoration​​. Moreover, the treatment demonstrated a paracrine effect in controlling hyperglycemia, body weight loss, and damage to organs like the pancreas, kidney, and liver, highlighting its systemic therapeutic benefits​ [119].

Another group purified EVs from high-glucose (HG)-stimulated Schwann cells to investigate and establish the DPN model. They hypothesized that the EVs from Schwann cells exposed to HG levels could speed up the onset of DPN by altering miR levels in dorsal root ganglia (DRG) neurons and their target proteins. In vivo, injecting HG EVs into the sciatic nerves of diabetic mice resulted in reduced nerve conduction velocity and the induction of mechanical and thermal hypoesthesia, symptoms characteristic of DPN. This treatment also led to a decrease in intraepidermal nerve fibers. The alterations in miR levels and target protein expression observed in vitro were also detected in the sciatic nerve tissue of these mice but not in their DRG tissue, suggesting a localized effect of the EVs [296]. This provides a potential new target for intervention in this common diabetic complication.

## Conclusion and future perspective

Research on EVs has burgeoned in recent decades, particularly their potential to enhance tissue regeneration [297, 298]. EV-based therapies hold significant promise for targeted delivery and uptake by specific tissues. EV-based tissue regeneration has been underscored by previous investigations; however, several critical issues should be addressed before clinical translation [298]. EVs storage instability negatively impact their quality and efficacy, while the complexity and low yields of the reported protocols for EV isolation hinder large-scale production [298].

EVs facilitate cellular communication through various paracrine and endocrine endocytic mechanisms [285], such as micropinocytosis, phagocytosis, clathrin-independent endocytosis, and clathrin-mediated endocytosis. Additionally, EV cellular interaction is governed by the expression of cell surface proteins and EVs proteins [28]. However, uncertainties persist regarding how EV cargo evades degradation once internalized by recipient cells [299]. Nevertheless, EVs offer a versatile approach for delivering bioactive payloads, affording potential alternatives to parental stem cell therapy, particularly through the application of stem cell-derived EVs for the treatment of incurable diseases. EV engineering aims to enhance targeting capabilities, enable the loading of various endogenous and exogenous agents, and modify surfaces to obtain the desired therapeutic effects. Various techniques, including EV surface engineering, parental cell preconditioning, and loading of exogenous materials, can enhance EV biological activity and address limitations associated with natural EVs. By incorporating targeting moieties, EV surface engineering through incorporating targeting moieties can improve tissue-specific targeting and therapeutic efficacy [300]. Various techniques, such as electroporation, acoustic degradation, drug loading, and acoustic degradation can be utilized to modify EVs to enhance their therapeutic capability. The application of osmotic pressure and cytochalasin B improves EV yield and their capacity for drug loading [301].

In this review, we explored the therapeutic potential of engineered EV tissue regeneration, focusing on bone, skeletal muscles, and PNS. Notably, EVs exhibit robust ROS scavenging capability, contributing to tissue repair. ROS is a double-edged sword with positive and negative facets. The ROS-scavenging function of EVs markedly promotes tissue regeneration, however, further investigations are warranted to elucidate the mechanisms underlying EV-mediated ROS modulation and its therapeutic implications. Future studies should also investigate the loading of antioxidant proteins or enzymes into EVs and elucidate specific cargos with robust antioxidant activity. Various in vivo verification of reported activities by EVs and the specific EV cargo that is involved in their antioxidant activity need to be elaborated on in further studies.

Despite progress in EV engineering, major hurdles remain for successful clinical translation. Large-scale production and modification of therapeutic EVs are essential, alongside comprehensive in vivo assessments of biosafety and toxicological effects. Long-term evaluation, encompassing factors such as toxicity, biocompatibility, immunogenicity, drug-controlled release, and metabolic capacity, is essential to establish rigorous biomedical standards for engineered EVs and ensure their safe clinical application.

## Data Availability

The review is based on the published data and sources of data upon which conclusions have been drawn can be found in the reference list.

## Abbreviations

EV: Extracellular vesicles
ROS: Reactive oxygen species
ELNV: Exosome-like nanovesicles
MVB: Multivesicular bodies
MVs: Microvesicles
EVs: Extracellular vesicles
MSC: Mesenchymal stem cell
OA: Osteoarthritis
PD: Parkinson’s disease
PNS: Peripheral nervous system
ECM: Extracellular matrix
PEG: Polyethylene glycol
Apt-Exos: Aptamer-functionalized exosomes
AMSC: Adipose-derived mesenchymal stem cell
UCMSC: Umbilical cord mesenchymal stem cell
BMSC: Bone marrow-derived stem cell
GMSC: Gastric stem cell
hiPSC: Human-induced pluripotent stem cells
AFSC: Amniotic fluid stem cell
GelMA: Methacrylated gelatin
AβOs: Amyloid-β-peptide
CHA: Coralline hydroxyapatite
GCS: Glycol chitosan
PU: Polyurethane
CPO: Calcium peroxide
MBG: Mesoporous bioactive glass
HA: Hydroxyapatite
SDF-1: Stromal cell-derived factor 1
FBS: Fetal bovine serum
SDT: Sonodynamic therapy
PlatinumNP: Platinum nanoparticle
NOX5: NAD(P)H oxidase 5
VSMC: Vascular smooth muscle cell
RAC: Human retinal astrocyte
tBHP: Tert-butyl hydroperoxide
GPX: Glutathione peroxidase
SIRT1: Stimulating silent information regulator 1
Nrf2: Nuclear factor-erythroid 2-related factor 2
HO-1: Heme oxygenase-1
VEGFR: Vascular Endothelial Growth factor
EtOH: Ethanol
Bax: Bcl-2 associated X protein
Bcl2: B-cell lymphoma 2
Atg12: Autophagy Related 12
TRPML1: Transient receptor potential mucolipin 1
mTOR: Mammalian target of rapamycin
GSSG: Oxidized glutathione
H_2_O_2_: Hydrogen peroxide
GSH: Glutathione
NAC: N-acetylcysteine
SOD: Superoxide dismutase
CAT: Chloramphenicol acetyltransferase.
6-OHDA: 6-hydrxydopamine
RT: Room temperature
fALS: Familial amyotrophic lateral sclerosis
RASA1: RAS P21 protein activator 1
PI3K: Phosphoinositide 3 kinase
AKT: Protein kinase B
eNOS: Endothelial nitric oxide synthesis
BIRI: Ischemia-reperfusion injury
TP53INP1: Tumor protein 53-induced nuclear protein 1
MDA: Malondialdehyde
GSH-Px: Glutathione peroxidase
NF-kB: Nuclear factor-kappa B
NLRP3: Nitrogen lipid regulator protein 3
AD: Alzheimer’s disease
TrxR1: Thioredoxin reductase 1
GLT1: Glutamate transporter 1
CCl4: Carbon tetrachloride
ERK: 1/2 Extracellular-regulated kinase 1/2
DDB: Bifendate
MnSOD: Manganese-containing superoxide dismutase
EXO-hCdH: Human chemically derived hepatic stem cells
GCL: Glutamate cysteine ligase
CPC: Cardiac resident mesenchymal progenitor cell
Irak: 1 Interleukin-1 receptor-associated kinase
Traf6: Tumor necrosis factor receptor-associated factor 6
Mpo: Myeloperoxidase
Smad4: Signaling effector mothers against decapentaplegic protein 4
NPC: Neural progenitor cell
AngII: Angiotensin II
AMI: Acute myocardial infarction
DMT1: Divalent metal transporter 1
MDA: Malondialdehyde
CSC: Cardiac stem cell
CaMKII: Calcium/calmodulin-dependent protein kinase II
MIR: Myocardial ischemia/reperfusion
IVDD: Intervertebral disc degeneration
NP: Nucleus pulposus
LRR: Leucine-rich repeat
PYD: domain Pyrin domain
CKD: Chronic kidney disease
PrPC: Prion protein
PTC: Peritubular capillary
HUVEC: Human Umbilical Vein Endothelial Cell
AKI: Acute kidney injury
NRK-52E: Renal tubular epithelial
HLEC: Human lens epithelial cell
CRTAC1: Cartilage acid protein 1
POI: Premature ovarian insufficiency
hGC: Human primary granulosa cell
SIRT4: Sirtuin4
TNBS: 2, 4, 6-trinitrobenzene sulfonic acid
iNOS: Inducible nitric oxide synthase
COX-2: Cyclooxygenase-2
PARP: ADP-ribose polymerases
CDK: Kidney of CKD patients
MAPK: Mitogen-activated protein kinase
WJMSC: Wharton’s Jelly-Derived Mesenchymal stem cell
CRTAC1: Cartilage acidic protein 1
TNBS: Trinitrobenzenesulfonic acid
PNS: Peripheral nerve system
DBD: Diabetic bone disease
rOB: Rat osteoblast
T2DM: Type 2 diabetes mellitus
DMD: Duchenne muscular dystrophy
HEK: Human embryonic kidney
MYMK: MyoMaker
MYMX: MyoMixer
M-CAD: M-Cadherin
DPN: Diabetic peripheral neuropathy
TLR4: Toll-like receptor 4
HG: High-glucose
NG: Normal glucose
DRG: Dorsal root ganglia
