# Correction: Engineering extracellular vesicles for ROS scavenging and tissue regeneration

**DOI:** 10.1186/s40580-024-00470-1

**Published:** 2025-01-27

**Authors:** Ahmed Abdal Dayem, Ellie Yan, Minjae Do, Yoojung Kim, Yeongseo Lee, Ssang-Goo Cho, Deok-Ho Kim

**Affiliations:** 1 Department of Stem Cell and Regenerative Biotechnology, Molecular & Cellular Reprogramming Center, Institute of Advanced Regenerative Science, Konkuk University, 120 Neungdong-ro, Gwangjin-gu, Seoul, 05029 Republic of Korea; 2 Department of Biomedical Engineering, Johns Hopkins University, Baltimore, MD 21205 USA; 3 R&D Team, StemExOne Co., Ltd., 307 KU Technology Innovation Bldg, 120, Neungdong-ro, Gwangjin- gu, Seoul, 05029 Republic of Korea; 4 Department of Mechanical Engineering, Johns Hopkins University, Baltimore, MD 21205 USA; 5 Department of Medicine, Johns Hopkins University School of Medicine, Baltimore, 21205 USA; 6 Center for Microphysiological Systems, Johns Hopkins University, Baltimore, MD 21205 USA; 7 Institute for NanoBiotechnology, Johns Hopkins University, Baltimore, MD 21218 USA; 8 Department of Neurology, Johns Hopkins University School of Medicine, Baltimore, MD 21205 USA

Correction to: Nano Convergence (2024) 11:24

10.1186/s40580-024-00430-9

The original publication of this article [1] contained errors in Funding section. The incorrect and correct Funding texts are listed with this correction article. The original article has been updated.

Incorrect:

**Funding**

This study was supported by the National Institutes of Health (NIH) grants, UH3TR003519, R01HL156947, R01HL164936, R01HL146436, R01HL164936, R01NS133965, R21AI168886 and UH3TR003271 (D.-H.K.); Program of the National Research Foundation of Korea (NRF) funded by the Korean Government (Ministry of Education, Science, and Technology), Grant number: 2019M3A9H1030682, as well as Korean Fund for Regenerative Medicine (KFRM) grant by the Korean government (the Ministry of Science and ICT and the Ministry of Health & Welfare) Grant number: 22B0502L1-01 (S.-G.C.). This paper was supported by the KU-Research Professor Program of Konkuk University, Seoul, South Korea (A.A.D.).

Correct:

**Funding**

This study was supported by the National Institutes of Health (NIH) grants, UH3TR003519, R01HL156947, R01HL164936, R01HL146436, R01HL164936, R01NS133965, R21AI168886 and UH3TR003271 (D.-H.K.). This study was supported by the Korean Fund for Regenerative Medicine (KFRM) grant funded by the Korean government (the Ministry of Science and ICT and the Ministry of Health & Welfare) Grant numbers: 22B0502L1-01 and 24A0203L1 (S.-G.C.). This paper was supported by the KU-Research Professor Program of Konkuk University, Seoul, South Korea (A.A.D.)”
